# Quantitative definition and monitoring of the host cell protein proteome using iTRAQ – a study of an industrial mAb producing CHO‐S cell line

**DOI:** 10.1002/biot.201500550

**Published:** 2016-06-22

**Authors:** Lesley M. Chiverton, Caroline Evans, Jagroop Pandhal, Andrew R. Landels, Byron J. Rees, Peter R. Levison, Phillip C. Wright, C. Mark Smales

**Affiliations:** ^1^ Centre for Molecular Processing and School of BiosciencesUniversity of KentCanterburyKentUK; ^2^ ChELSI Institute, Department of Chemical and Biological EngineeringUniversity of SheffieldSheffieldUK; ^3^ Pall Life SciencesPortsmouthUK

**Keywords:** Bioprocessing, Chinese hamster ovary (CHO), Host cell proteins, iTRAQ, Monoclonal antibody

## Abstract

There are few studies defining CHO host cell proteins (HCPs) and the flux of these throughout a downstream purification process. Here we have applied quantitative iTRAQ proteomics to follow the HCP profile of an antibody (mAb) producing CHO‐S cell line throughout a standard downstream purification procedure consisting of a Protein A, cation and anion exchange process. We used both 6 sample iTRAQ experiment to analyze technical replicates of three samples, which were culture harvest (HCCF), Protein A flow through and Protein A eluate and an 8 sample format to analyze technical replicates of four sample types; HCCF compared to Protein A eluate and subsequent cation and anion exchange purification. In the 6 sample iTRAQ experiment, 8781 spectra were confidently matched to peptides from 819 proteins (including the mAb chains). Across both the 6 and 8 sample experiments 936 proteins were identified. In the 8 sample comparison, 4187 spectra were confidently matched to peptides from 219 proteins. We then used the iTRAQ data to enable estimation of the relative change of individual proteins across the purification steps. These data provide the basis for application of iTRAQ for process development based upon knowledge of critical HCPs.

See accompanying commentary by Kelvin Lee DOI 10.1002/biot.201600223

**Abbreviations**
CHO: Chinese hamster ovary
CV: column volume
2D‐DIGE: two dimensional‐difference gel electrophoresis
DSP: downstream processing
ELISA: enzyme‐linked immunosorbent assay
HC: antibody heavy chain
HCCF: harvest cell culture fluid
HCP: host cell protein
iTRAQ: isobaric tag for relative and absolute quantitation
LC: antibody light chain
LC‐MS/MS: liquid chromatography coupled mass spectrometry
mAb: monoclonal antibody

## 1 Introduction

Monoclonal antibodies (mAbs) constitute the largest class and number of biotherapeutic recombinant proteins currently on the market 1. The Chinese hamster ovary (CHO) cell is preferentially utilized for the production of recombinant mAbs due to their ability to produce correctly folded and assembled mAb with human‐like glycans 2, 3, 4. The mAbs are secreted into the harvest cell culture fluid (HCCF) during fermentation and recovered from this during downstream processing to separate the required mAb from process (e.g. host cell proteins [HCPs], DNA, RNA, lipids and other cellular derived material) and product (e.g. mAb fragments, aggregates) impurities 5. HCPs found in the HCCF can be derived either from secreted endogenous CHO proteins or released into the cell culture supernatant upon cell lysis, including during collection of the harvest material. A number of studies have suggested that cell viability at harvest is a critical aspect in determining the number and presence of those HCPs present in the downstream processing feedstock (see 6, 7 for reviews). Whilst there is no precise specification for total HCP content in final biotherapeutic preparations, with preparations being judged on a case‐by‐case basis 8, generally the literature, industry and regulatory authorities work towards HCP limits of 1–100 ppm (1–100 ng HCP per mg biotherapeutic protein) in the final product 9. HCPs remaining in biotherapeutic recombinant proteins potentially present a risk to the patient 9, 10, 11, the major potential risk suggested to be that of immunogenicity 12.

A number of studies have now reported on the monitoring of HCPs during process development of biotherapeutic proteins, including mAbs, in order to inform about the development of, and validate, such processes. The downstream purification of mAbs from CHO cell culture harvest fluid is heavily reliant upon depth filtration and chromatographic approaches to remove process and product impurities. A ‘typical’ downstream purification process for mAbs includes an initial Protein A chromatography step, often followed by a low pH hold viral inactivation step, followed by one or two more polishing steps and an additional viral filtration step before the product is concentrated and formulated for use in the clinic 13. For mAbs, key studies have now been published showing that the specific interaction of HCPs with the product during Protein A affinity chromatography results in particular HCPs co‐eluting with the mAb 14. Therefore, the product makes a significant contribution to defining the HCPs that co‐elute with the mAb 15.

The dynamic range of HCP concentrations in the cell culture harvest material, and throughout downstream processes, means that not any one technology is able to detect and monitor all those HCPs present. Multi‐analyte ELISA is the current workhorse approach adopted throughout the industry to measure and monitor HCP content/concentration due to its high throughput nature, selectivity and sensitivity 9, 16. However, there are limitations to the ELISA approach, therefore the use of orthogonal methods to support process development and validation is recommended 9. The major orthogonal approaches that have emerged to monitor/measure HCPs are 2D‐PAGE 17, particularly 2D‐DIGE 18, 19, and mass spectrometry 14, 20, 21, 22, 23, 24. These approaches give information on the specific nature/identification of those HCPs present during up‐stream and downstream processing, allowing clearance and risk to be assessed on the nature of those HCPs present, rather than on the combined concentration of HCPs 7. Indeed, a recent report suggests that the application of LC‐MS/MS approaches could be applied to overcome limitations of the ELISA and 2D‐PAGE approaches for HCP monitoring 16. Such an approach has been applied to the profiling of downstream HCPs in therapeutic peptibodies expressed in *Escherichia coli,* demonstrating how process changes influenced subsequent residual HCP content and makeup 25.

The use of combined orthogonal approaches, including mass spectrometry, to monitor HCPs from CHO cell cultures has now been reported by a number of groups. For example, Pezzini et al. 26 demonstrated how conditions for ‘optimal’ mixed mode chromatography purification of mAbs from CHO cell culture harvest material can be determined by utilizing Design of Experiment modeling approaches combined with mass spectrometry analysis to identify those HCPs co‐purifying with the target mAb. The differences in selectivity and efficiency of classical versus multimodal cation exchange chromatography for mAb purification with respect to those HCPs retained in the mAb fraction have also been demonstrated by mass spectrometry 22. A comparison of the HCP profile of three null CHO cell lines using ELISA, 2D‐PAGE and LC‐MS/MS approaches indicated that the HCPs in different feedstocks for downstream processing were not as diverse as might have been expected 16. Indeed, reports suggest that it is a subset of the total HCP profile present in CHO cell culture supernatants that are more difficult to purify or remove during downstream processing, as they interact with chromatography media and/or co‐purify with the target product 27. Valente et al. used a combination of 2D‐electrophoresis and shotgun proteomic approaches to demonstrate that the cell age impacts upon the extracellular CHO HCP profile, identifying specific proteins whose expression profile changes with culture time 28. Zhang and colleagues further demonstrated the potential of mass spectrometry for monitoring HCPs during process change, tracking HCPs from the HCCF through to Protein A eluate and further downstream, identifying around 500 HCPs in the HCCF, following these until no HCPs were identified in the final cation‐exchange chromatography eluate 24.

Here, we use iTRAQ non‐gel based LC‐MS/MS proteomic profiling to enhance the coverage of HCPs detected beyond standard 2D‐PAGE 8, and apply quantitative mass spectrometry to define the harvest supernatant HCP proteome of a mAb producing CHO‐S host cell line, and follow the HCP profile during a standard downstream mAb purification following expression in a fed‐batch 100 L wave bioreactor. We have used this approach to characterize and profile the HCPs in the harvest cell culture fluid (HCCF), and to follow the fate of each HCP throughout downstream processing (DSP) using a typical purification process. iTRAQ was implemented in two workflow formats: to analyze DSP by Protein A chromatography (six sample analysis) and Protein A followed by additional chromatographic cation and anion exchange steps (eight sample analysis). These data indicate that the majority, if not all, HCPs detectable in the HCCF are detectable throughout the whole of the downstream process examined, albeit at very much reduced amounts. The enrichment of specific HCPs as a percentage of the total throughout the downstream process is also evident.

## 2 Materials and methods

All materials and reagents were sourced from Sigma‐Aldrich, UK, unless otherwise stated.

### 2.1 Cell culture of a model CHO‐S mAb producing cell line and preparation of the HCCF for downstream processing.

The model CHO‐S mAb producing cell line had previously been engineered to stably express a model IgG1 mAb against HER2 (human epidermal growth factor receptor 2) and was cultured at Pall Life Sciences (Portsmouth, UK). For the 100 L single‐use rocker bioreactor experiment, the culture was inoculated with 2.3 × 10^5 ^viable cells per mL from an exponentially growing seed train culture in CD1000 media (BD Biosciences) supplemented with 30% CHO CD Efficient Feed™ A AGT™ (Invitrogen), 1.47 g/L sodium bicarbonate, 8 mM L‐glutamine, 0.5% w/v Boost 4 (Hyclone) and 1.0 g/L pluronic F86. The cell number and viability were monitored throughout culture using a Vi‐Cell instrument (BD). At 374 h of culture (viability 52%), the harvest cell culture fluid (HCCF) was collected by centrifugation.

### 2.2 Protein A chromatography

HCCF was loaded onto a prepacked HiTrap^®^ MabSelect™ SuRe™ column (1 mL, GE Healthcare, Little Chalfont, UK) with the pressure threshold set at 0.5 MPa. The column was equilibrated with 10 column volumes (CVs) of 20 mM sodium phosphate pH 6.8 at a flow rate of 1 mL/min and the sample then loaded at 2.5 mL/min and the flow‐through collected. The column was washed with 5 CVs of 20 mM sodium citrate, pH 5 and the mAb then eluted with 3 CVs of 20 mM sodium citrate, pH 3.5 and the eluate collected. For HCCF subjected to Protein A affinity chromatography only, samples were run in 5 × 10 mL lots through the column, and then all eluate fractions combined. For the experiment analyzing HCCF, Protein‐A eluate, and then subsequent post‐cation and anion exchange material, 150 mL of HCCF was subjected to Protein A affinity purification in 15 × 10 mL runs with the subsequent eluates combined after each step. The mAb containing fractions were then pooled for further downstream processing, with a sample of the pooled fractions retained for subsequent HCP analysis.

### 2.3 Cation exchange chromatography

The combined pooled volume of post‐Protein A eluate (45 mL) was loaded onto a 5 mL prepacked S HyperCel™ column (Pall Life Sciences, Portsmouth, UK) witha pressure threshold of 0.5 MPa and a flow rate of 1 mL/min. The column was equilibrated with 10 CVs of 20 mM sodium citrate, pH 3.75 and the sample then loaded onto the column at 2.5 mL/min. The column was washed with 5 CVs of 20 mM sodium citrate, pH 3.75. The mAb was eluted with 6 CVs of 20 mM sodium phosphate, pH 7, followed by a 5 CV 10 min gradient from 100% 20 mM sodium phosphate, pH 7, to 100% 20 mM sodium phosphate/1 M sodium chloride at 2.5 mL/min. The eluate was kept for anion exchange chromatography.

### 2.4 Anion exchange chromatography

Acrodisc Mustang Q, 1 mL format (Pall Life Sciences, Portsmouth, UK) was used for anion chromatography of the post‐cation exchange eluate. Preparation of the membrane disc for use was undertaken by washing with 4 mL of 1 M sodium hydroxide followed by washing with 4 mL of 1 M sodium chloride. The sample was then passed through the column and the flow‐through collected.

### 2.5 2D‐PAGE analysis of HCPs

2D‐PAGE analysis was undertaken as previously described 6, 29, 30.

### 2.6 HCP ELISA

HCP concentrations were determined in samples using the commercially available ELISA kit from Cygnus Technologies. The concentration of HCPs present in samples was then determined from a standard curve as previously described 30.

### 2.7 Sample preparation for iTRAQ proteomic analysis, HILIC‐HPLC fractionation, mass spectrometry analysis, proteomic data identification, and proteomic data analysis

Mass spectrometry sample preparation, experimental setup and data analysis were all undertaken following standard procedures as described in detail in the Supporting information, Methods file S1. Due to the size and cost of the 100 L wavebag experiments, technical replicates were analyzed by the iTRAQ proteomic approach.

### 2.8 Pathways analysis

Pathway analysis was performed using UniProtKB for Gene Ontology analysis. The freeware software Panther (www.pantherdb.org) was used for classification of identified proteins into functional classes.

### 2.9 Heatmaps

Heatmaps were generated using an R software package (see Warnes, G. R., Bolker, B., Bonebakker, L., Gentleman, R. et al., gplots: Various R programming tools for plotting data. R package version 2.12.1; http://CRAN.R‐project.org/package=gplots2013).

## 3 Results

### 3.1 Recombinant mAb production, purification and determination of HCP concentrations throughout the downstream process by commercial HCP ELISA, SDS‐ and 2D‐PAGE

**Figure 1:**
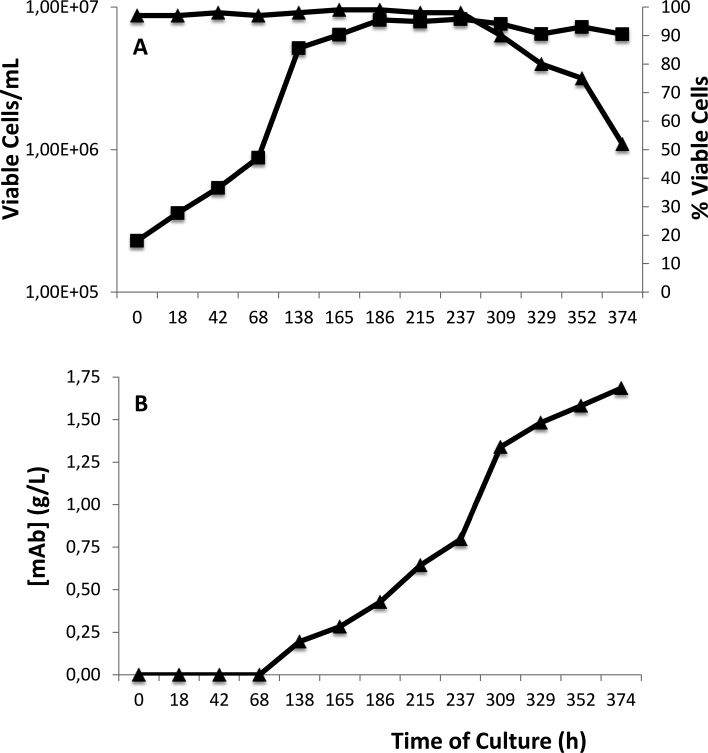
Viable cell number, culture viability and antibody concentration data during the CHO‐S mAb producing cell line fed‐batch 100 L single‐use rocker culture. (**A**) viable cell numbers (squares, ▪) and associated cell viability (triangles, ▴) across the fermentation. (**B**) mAb concentration in the cell culture supernatant throughout culture. The harvest cell culture fluid (HCCF) was harvested for analysis of HCPs at 374 h.

We used a mAb expressing CHO cell line, cultured in a 100 L Single‐Use rocker bioreactor, as a model system to investigate the CHO‐S host cell protein proteome and how this changes through a standard mAb chromatographic downstream process. As described in the introduction, whilst the required mAb is secreted into the culture supernatant, endogenous CHO proteins can also be released via secretion out of the cell or during lysis/breakage of cells during culture and/or harvesting. Here, we applied iTRAQ proteomic profiling as a discovery tool to provide a broad quantitative analysis of the CHO HCP proteome and how this changes throughout a standard mAb downstream process. The HCCF from our model system was harvested after 374 h of culture when culture viability in the wavebag began to decline (Fig. 1A). The antibody concentration in the HCCF at this time was determined as 1.69 g/L (Fig. 1B), with the cell specific productivity during exponential growth phase, being 21 pg/cell/day.

**Figure 2:**
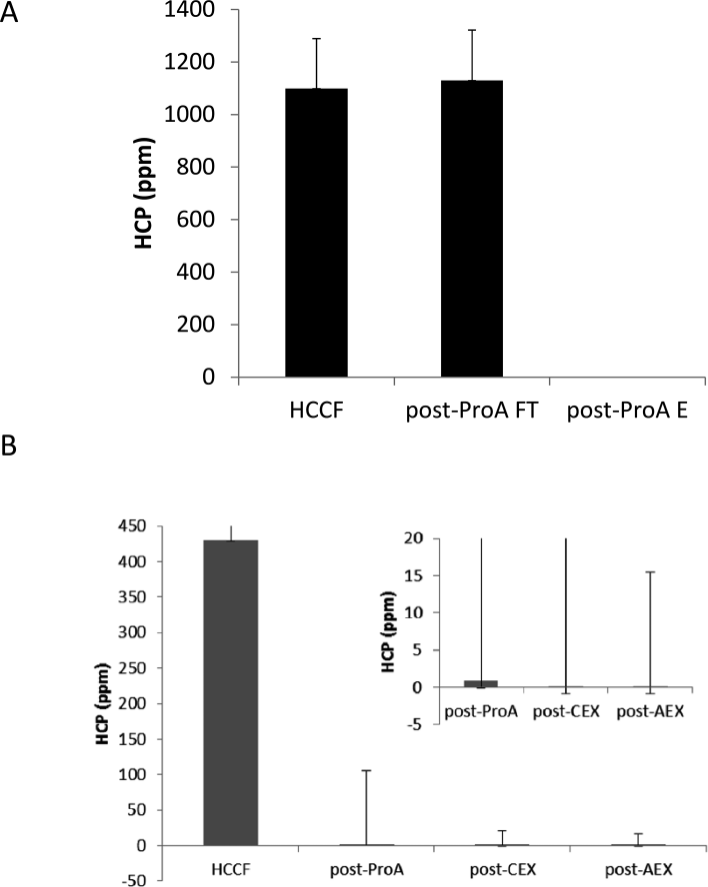
HCP analysis determined by ELISA of HCCF and samples during DSP. HCP concentrations were determined using commercially available ELISA for (**A**) HCCF, Protein A flow‐through, Protein A eluate, and (**B**) HCCF and eluates from sequential processing by Protein A, Cation exchange and anion exchange steps. Data are ± standard error of mean, *n* = 2 technical replicates.

**Figure 3:**
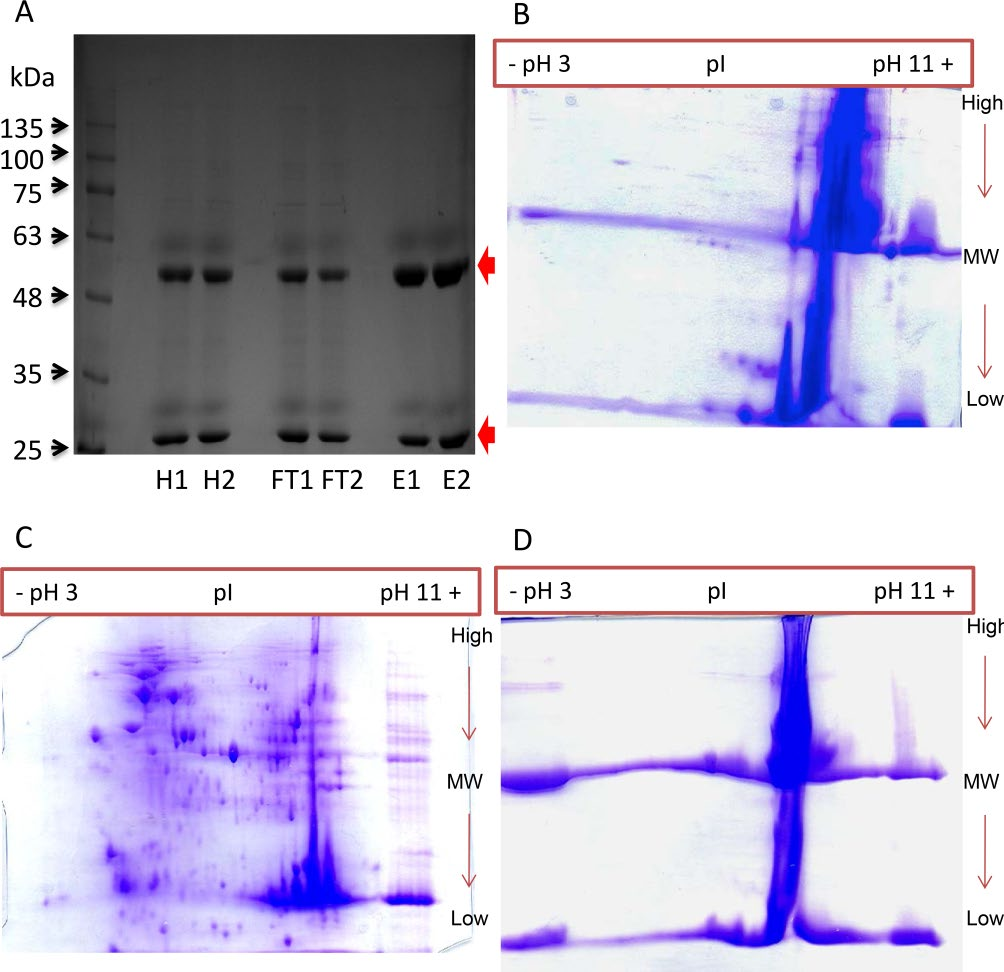
Reduced PAGE analysis of protein profiles of the HCCF of the CHO‐S mAb producer, Protein A eluate and post‐Protein A flow‐through. (**A**) 1D SDS‐PAGE Gel (two replicates of each condition, 5 µg total protein per sample loaded) analysis of samples. H, harvest; FT, Protein A flow through; E, Protein A elute. (**B**–**D**) 2D‐PAGE analysis of 100 µg of total protein from the HCCF (**B**), Protein A flow‐through (**C**) and Protein A eluate (**D**) material visualized using Coomassie Blue stain. Red arrows indicate the presence of heavy and light chains at 50 kDa and 25 kDa respectively.

After collection of the HCCF, we subjected this to a typical mAb downstream chromatographic purification process for subsequent HCP and proteomic analysis. Two purifications of the HCCF material were undertaken for two different iTRAQ HCP profiling experiments. For the first experiment, the HCCF was subjected to an initial primary purification step, consisting of a Protein A affinity chromatography step, and the flow‐through and eluate collected. The second experiment analyzed HCCF and samples from three sequential downstream processing steps: eluates from Protein A and cation exchange chromatography and the flow‐through from anion exchange. Samples were also stored and prepared for HCP ELISA and 2D‐PAGE analysis, the results of which are shown in Figs. 2 and 3. A total of 75 µg of total protein from each fraction was analyzed by iTRAQ‐based protein profiling with the workflows shown in Supporting information, Fig. S1. The commercial HCP ELISA methodology is based upon immune‐detection using anti‐HCP antibodies. The HCP ELISA data are shown for the six sample and eight sample experiments in Fig. 2. Figure 2A shows the HCP concentration, as determined from the ELISA data in the HCCF, and, subsequently, that in the Protein A flow‐through and eluate fractions. The concentration of HCP in the HCCF is approximately equivalent to that of the mAb, meaning there is an approximate 50:50 mix of mAb and HCP in the cell culture supernatant material. Almost all of the HCPs (>99%) were removed by the Protein A chromatography step as determined by the HCP ELISA, with the majority of HCPs being found in the flow‐through fraction. The concentration of HCP in the eluate was <10 ppm as determined using the commercially available HCP assay (Fig. 2A). This was also observed in the samples generated for the eight sample experiment, with the subsequent cation and anion exchange steps used in this experiment making a small, but insignificant, difference to the total amount of HCP present in these samples (Fig. 2B). However, these data provide no information about which HCPs remained as the purification process progressed, and whether all HCPs were reduced in concentration, or if some were concentrated along with the mAb.

Reduced SDS‐PAGE and 2D‐PAGE analysis was then undertaken on the HCCF, Protein A flow‐through and Protein A eluate samples generated from the six sample experiment (Fig. 3). These analyses again confirmed the enrichment of the recombinant mAb heavy and light chains in the Protein A eluate, and that they were also both present in the flow‐through fraction (Fig. 3A). 2D‐PAGE analysis clearly showed the concentration of the heavy and light chain in the Protein A eluate and depletion of HCPs in this fraction, with the majority of HCPs observed in the Protein A flow‐through fraction. In each case, the heavy and light chain polypeptides are the most intense bands/spots on the gel, at 50 kDa and 25 kDa (Fig.s 3B–D) respectively, as the gels are run under reducing conditions. The presence of other protein spots on the gels likely correspond to HCPs, visible in the HCCF and post‐Protein A flow‐through. However, these spots are not visible using Coomassie stain in the post‐Protein A eluate, and mAb dominates and obscures the gel image. This justifies the need for alternative analyses for the presence of HCPs co‐enriching with mAb in such samples. Although western blot analysis of 2D‐PAGE can be undertaken using anti‐HCP antibodies, this does not allow identification of specific HCPs, and, hence, we applied the non‐gel based iTRAQ proteomic approach as a discovery tool to monitor and identify HCPs present in the HCCF and throughout the downstream purification process.

### 3.2 iTRAQ proteomic profiling of CHO HCPs throughout a standard mAb downstream purification process

The proteome at each stage of the downstream purification process was analyzed (in technical replicates) using quantitative proteomics. In this regard the approach we have taken mimics that for a ‘production run’ generating clinical material, where each batch of product generated is done in a single run after which the HCP profile is required to be detailed without the ability to analyze biological replicates. The iTRAQ approach used here was favored over gel‐based methods, as it has the capacity to identify proteins based on their primary sequence – rather than mass or isoelectric point – at a far higher level of sensitivity 7. It also complements the HCP ELISA experiment, as it has the capacity to detect proteins that do not elicit an immune response. For quantitative proteomic analysis, the isobaric tagging strategy iTRAQ was used. This methodology enables simultaneous identification and relative quantification of proteins, in multiplex format, of up to eight samples within a single experimental run 31.

As described in the Methods section, 8‐plex iTRAQ was implemented in two workflow formats, employing either six samples (with two labels reserved for noise modeling) or eight samples, as demonstrated in (Supporting information, Fig. S1). The six sample iTRAQ experiment was directed to analyze technical replicates of three samples, which were HCCF, Protein A flow‐through and Protein A eluate (Supporting information, Fig. S1A). The eight sample format was used to analyze technical replicates of four sample types: HCCF compared to Protein A eluate and subsequent cation and anion exchange purification of the Protein A eluate (Supporting information, Fig. S1B). The data were processed for protein identification and relative quantification. The list of proteins identified are reported in Supporting information, Tables S1 and S2.

In the six sample iTRAQ experiment, 8781 spectra were confidently matched to peptides from 819 proteins (including the mAb chains). Across both the six and eight sample experiments 936 proteins were identified. In the eight sample comparison, 4187 spectra were confidently matched to peptides from 219 proteins. The reduced number of identifications in the eight sample iTRAQ, relative to the six sample experiment, occurred due to mAb dominance in the sample, leading to a stochastic reduction in the identification of HCP peptides. This resulted in 83.4% of the peptide spectral match (PSM) being matched to mAb derived peptides; for comparison, mAb derived peptides contributed only 47% of PSM in the six sample analysis. The same replicates of HCCF were used on both experiments, providing a common sample to link analyses. Of the 219 proteins identified in the eight sample iTRAQ, 100 were common to the six sample iTRAQ; all 219 proteins identified to a three or more unique peptide confidence in the eight sample analysis were present in the six sample comparison. Interestingly, proteins identified and retained include proteases such as cathepsins and serine proteases, such molecules having previously been shown to result in degradation of mAbs after purification 32, 33, 34, and thioredoxin reductase, which has been identified as playing a major role in the reduction of intact mAb in cell culture supernatants 35, 36.

### 3.3 Global quantification analysis of mAb and HCP

**Figure 4:**
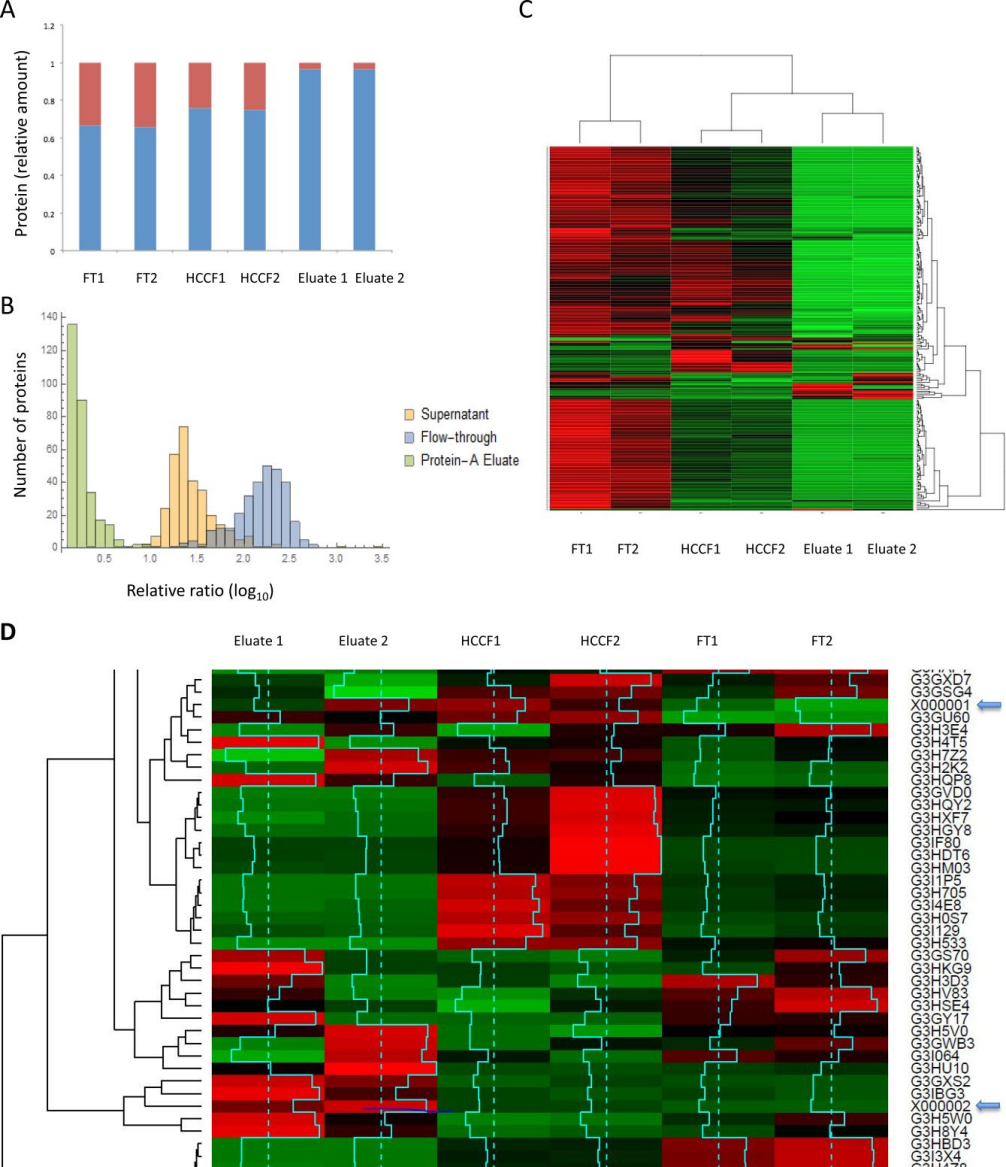
Quantitative proteomic analysis of Protein A purification. (**A**) Stacked bar chart of relative amounts of mAb and HCP based on iTRAQ reporter ion intensity values as a proportion of the total. HCP and mAb shown in red and blue respectively. (**B**) Relative distribution of HCP during Protein A chromatography, protein ratios are calculated from individual peptide intensity values divided by the mean of all intensities. (**C**) Two color heatmap: rows represent individual proteins identified and relatively quantified by three or more unique peptides, columns represent the individual samples. The intensity scale is brightest green, black, brightest red, where green indicates relative decrease and red increase in protein level. (**D**) Heatmap where rows represent individual proteins identified and relatively quantified by three or more peptides, columns represent the individual samples. Intensity scale green, black, red, with red indicating increase and green decrease. Data are shown for the HCCF starting material and the Protein A eluates. Protein heavy (X000002) and light (X000001) chains are highlighted with blue arrows. The row z score (turquoise) is calculated by centering the rows, scaled by subtracting the mean of the row from every value and then dividing the resulting values by the standard deviation of the row.

**Figure 5:**
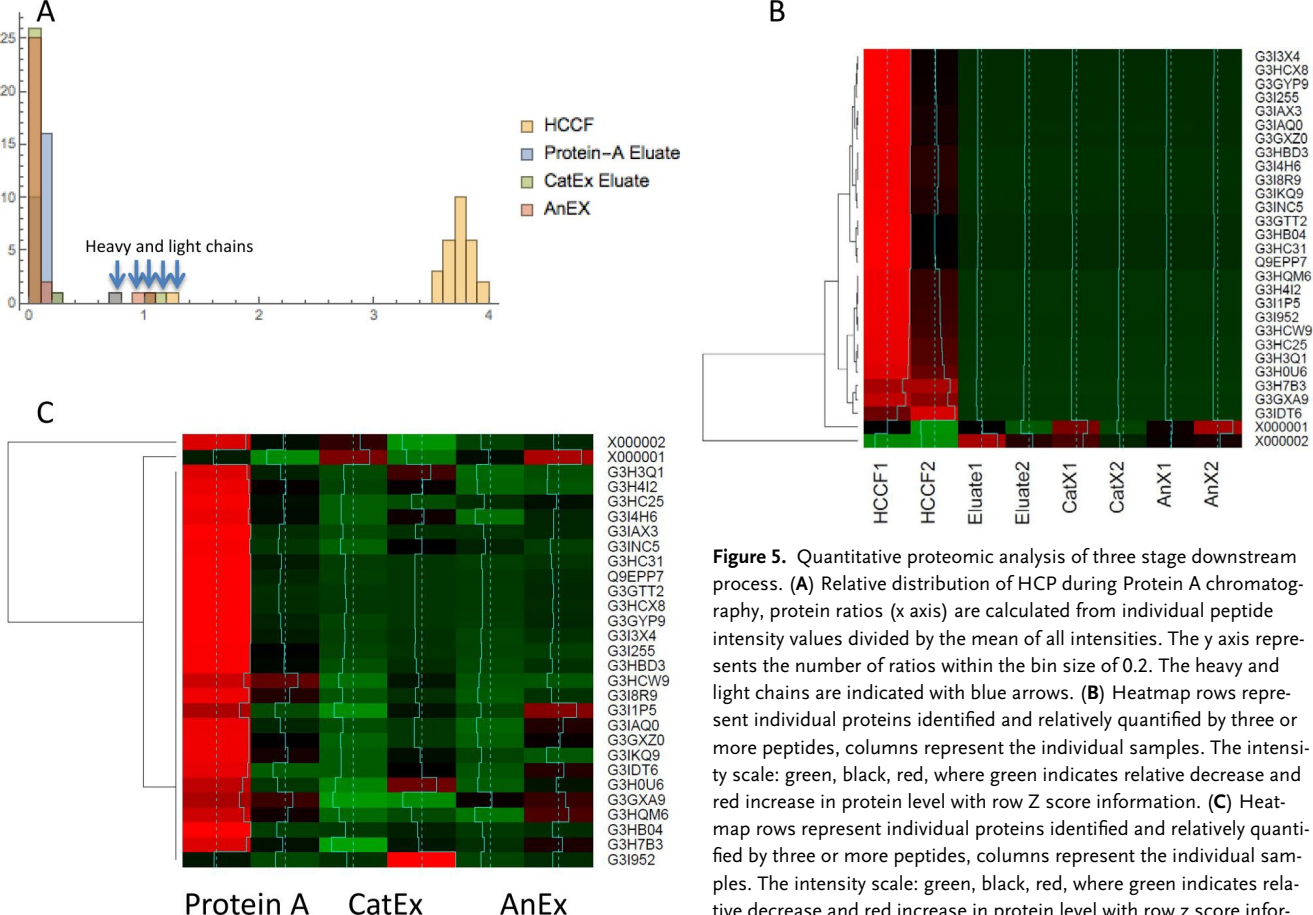
Quantitative proteomic analysis of three stage downstream process. (**A**) Relative distribution of HCP during Protein A chromatography, protein ratios (x axis) are calculated from individual peptide intensity values divided by the mean of all intensities. The y axis represents the number of ratios within the bin size of 0.2. The heavy and light chains are indicated with blue arrows. (**B**) Heatmap rows represent individual proteins identified and relatively quantified by three or more peptides, columns represent the individual samples. The intensity scale: green, black, red, where green indicates relative decrease and red increase in protein level with row Z score information. (**C**) Heatmap rows represent individual proteins identified and relatively quantified by three or more peptides, columns represent the individual samples. The intensity scale: green, black, red, where green indicates relative decrease and red increase in protein level with row z score information. The mAb heavy and light chains are present in the top rows of the heatmap.

By normalizing the protein quantitations by channel sum, each iTRAQ label was scaled so that the sum of all reporter ion intensities were evaluated as equal, enabling estimation of the global concentrations of HCPs and mAb within the sample. As expected, this method showed reducing global levels of HCP relative to mAb during Protein A chromatography in both iTRAQ experiments (Figs. 4A, 4B and 5A). The relative change of individual proteins across the purification steps is presented in heatmap format (Figs. 4 and 5).

An interesting observation from the 8‐plex iTRAQ data was an alteration in the ratio of heavy (HC) to light (LC) chains of the mAb (Supporting information, Table S3). This was calculated by balancing the HC and LC ratios to be even at the reference label – in this case in the Protein A eluate – and then observing how the relative quantifications changed after each progressive purification step. In the HCCF samples, there is a 2:1 ratio of LC to HC mAb. However, this balances out after Protein A chromatography. The change from the HCCF to the Protein A eluate is not surprising, as this step should remove free LC and LC dimers that are present in the HCCF – resulting from the Protein A column binding of the HC species only (note that there is no Protein A flow‐through in this experiment, where the LC would be observed). The progressive reduction in the ratio of HC to LC in the following chromatography steps may indicate the removal of un‐bound HC, or could be indicative of degradation of the mAb complex.

### 3.4 Quantitative analysis of HCPs during downstream purification

As detailed in the Methods section, we approached the data analysis in two stages. HCPs detectable above the noise‐level at each stage of purification were determined. The mean iTRAQ ratio was determined if the relative amount of protein had reduced/increased as a consequence of DSP. Finally, we merged these two analyses to generate three lists of proteins for each purification step: proteins that were above the limit of detection, proteins that were either preferentially retained or decreased relative to the previous stage (Supporting information, Table S4). This analysis focused on proteins with data from three or more unique peptides in order to ensure robust quantification data.

In the six sample experiment, all proteins were detectable in the Protein A flow‐through, with 86 HCPs at detectable levels in the Protein A eluate. Of these 86 detectable proteins, two were preferentially retained, i.e. increased relative to HCCF starting material, and these were Proteasome subunit alpha type and Ubiquitin activating enzyme E1 (Uniprot accession numbers G3I9G7 and G3IBG3 respectively). In the eight sample experiment, in addition to the mAb, six HCPs proteins were detectable in the Protein A eluate, with five in common to those observed with the six sample experiment directed to Protein A purification. These proteins were Peroxiredoxin‐1, fructose bisphosphate aldolase, 78 kDa glucose regulated protein, pyruvate kinase and Cathepsin Z (Uniprot accession numbers G3GYP9, G3I3X4, G3I8R9, G3IAX3, Q9EPP7). Strikingly, Peroxiredoxin‐1, a redox regulatory protein (Uniprot accession number G3GYP9) was detectable in the anion exchange flow‐through. Further analysis revealed that this protein was also present in the cation exchange eluate, identified and relatively quantified with two unique peptides. The data suggest that Peroxiredoxin‐1 is retained during the three stage DSP, but its level was decreased relative to the Protein A eluate.

## 4 Discussion

The International Conference on Harmonisation Guideline Q11 on the development and manufacture of drug substances 2012 suggests HCPs are a critical quality attribute (CQA) 37. Although there have been a number of reports that identify and monitor HCPs during the bioprocessing of CHO produced biopharmaceuticals, the majority of approaches utilized do not allow for the identification of specific individual HCPs, or follow their fate during an entire downstream process. This makes a thorough risk‐based analysis of the HCP component of the end product difficult, and the concentration of HCPs alone may mask small amounts of high‐risk proteins, such as cytokines that are secreted by CHO cells 38.

In order to address this, a number of studies on the specific identification of HCPs throughout culture and during different aspects of the downstream processing of recombinant protein molecules (largely mAbs) produced in CHO cells 7 now have been reported. For example, a previous study investigating CHO HCPs that are difficult to remove, used iTRAQ and 2D‐PAGE during prolonged cultures of CHO cells and identified 92 HCPs that showed changes in expression across 500 days of culture over which the HCP profile was investigated, with 92% of these detected using the iTRAQ shotgun proteomics approach, and only 18% by the 2D‐PAGE approach, whilst 11% were detected by both approaches 28. We, therefore, applied an iTRAQ approach to determine the HCP profile in the HCCF of a 100 L CHO mAb culture and followed this during a subsequent 3‐step downstream purification process.

We identified 819 proteins in the HCCF using iTRAQ analysis, including many of the proteins previously identified in other studies. For identification of proteins, we applied a threshold of a minimum of three peptides per protein as described in the Methods section and Supporting information for identification confidence and to ensure that quantification statistics would be reliable. We note that this would certainly increase false negatives, but we wanted a balance between quantification reliability and the loss of a small amount of information on potentially interesting HCPs that might have been identified by one or two peptides, hence reducing the number of proteins we identified in the HCCF and subsequent downstream processing steps. We also note that as mass spectrometry instruments continue to develop so does sensitivity, and with this the number of proteins identified would be expected to increase but potentially the reproducibility of identifying lower abundance proteins may decrease. This should be considered when using any mass spectrometry approaches whilst for iTRAQ specifically the reported dynamic range of iTRAQ technology and the potential signal‐to‐noise limitations was described previously by ourselves 39.

Other reported LC‐MS/MS studies of CHO HCPs have identified about 1000 proteins across supernatant samples from three null cell lines 16, whilst Zhang and colleagues used two‐dimensional liquid chromatography/mass spectrometry to identify approximately 500 HCPs in the cell culture fluid of a mAb producing cell line, suggesting that the limit of detection for an individual HCP was ∽13 ppm 24. Interestingly, Yuk et al. concluded that the difference in the HCP profile (and, therefore, the feedstock entering a potential downstream process) between the supernatant of different null cell lines was not as diverse as might be expected 16 and, hence, the DSP and target product is pivotal in determining those HCPs that remain at the end of any subsequent downstream process. However, others have shown that the HCP profiles and amounts do change with culture viability 29 that can influence the final HCP profile. It should be noted that the difficulty in recovering HCPs from cell culture supernatant is not trivial and reports have shown that differences in the approaches taken to prepare samples can influence the subsequent results and coverage of HCPs obtained 27.

We then applied the iTRAQ analysis to follow the fate of the CHO HCPs during a subsequent model mAb chromatographic purification process. Previous reports have shown that LC‐MS/MS approaches detected 19 HCPs from a mAb preparation when combined with an enrichment strategy, as compared to one HCP without the enrichment strategy 20. Zhang et al. also used mass spectrometry approaches to monitor HCPs in a Protein A eluate pool and, in some cases, beyond, reporting that no individual HCPs could be detected in the final cation‐exchange chromatography eluate pools of their purification process 24. We initially utilized Protein A affinity followed by cation exchange chromatography, which is often used in a mAb purification process, in a bind and elute mode, to remove remaining product aggregates, HCPs, DNA and leached Protein A. Anion exchange was used as a final polishing step, operated in flow‐through mode, whereby the mAb product does not bind the resin but impurities such as HCPs and DNA do. It is well documented that specific HCPs interact with target mAb molecules and Protein A resins and so are retained or co‐purified with the target molecule, and a number of specific HCPs that associate with resins and mAbs have been identified (e.g. 15, 40, 41, 42, 43, 44). As the purification proceeds, the concentration of total and, thus of individual, HCPs is dramatically reduced and this is set against a background where the product mAb concentration may be in the 10–100s mg/mL. Despite this, we could detect the presence of HCPs throughout the DSP investigated, even at the end of the three‐step purification process. As reported in other studies, the majority of the detected HCPs were a subset of intracellular proteins. However, in contrast to other studies (e.g. 24), we were able to detect these throughout the purification process. Our results, as emphasized by the heatmaps that track the fate of HCPs throughout the purification process (Figs. 4 and 5), show that the chromatographic process was successful in reducing the total amount, but not all, HCPs.

Our analyses, therefore, show that there is a dynamic range of HCPs present in the HCCF of our model CHO‐S mAb producing cell line and that the majority of the HCPs are present throughout the purification process. This was initially surprising, as the commercial HCP ELISA suggested the clearance or removal of the majority of HCPs. The iTRAQ data give some indication as to those HCPs that are likely to be present in the highest amounts at the end of the purification process. Those HCPs persisting or remaining at higher concentrations at the end of the process might be considered critical HCPs that should be carefully monitored and risk assessed. However, simply being present is not sufficient to classify these proteins as critical, as many doses of therapeutic protein have now been administered without reported adverse effects directly related to the presence of specific HCPs. Additional information on the specific HCPs should be considered, such as their potential immunogenicity, using approaches to predict immunogenicity 12 or identifying components that may be highly active in the body at low concentrations, such as cytokines 10. HCPs that may be detrimental to the target recombinant product itself may also be identified and, in this regard, it is of note that cathepsins and serine protease HCPs were identified in the downstream process, each class of which has been reported in the literature to be problematic and have resulted in specific target product degradation 32, 33, 45.

The data we present here provide a comprehensive mapping of the CHO HCP at harvest and during a subsequent downstream process. Such data sets provide knowledge of those HCPs that are removed or reduced in amounts via specific chromatographic steps and those that are retained, either via interaction with the resin, or the mAb itself. By profiling the CHO‐S HCP proteome at harvest, and following the fate of these HCPs through downstream processing, the results of this study can be used to help ‘design’ new purification approaches trying to reduce specific HCPs and to design assays specific for those critical HCPs. A number of studies are now emerging demonstrating how the HCP profile of sister clones can be very similar 16, 17, and even in null cell lines of different CHO lineages there are remarkable similarities, suggesting that the findings from this study in CHO‐S are likely to have relevance to other CHO systems. Those critical HCPs will largely be product specific, however the development of new, rapid, analytical technologies based upon the knowledge of critical HCPs would be a step forward in the development of bioprocesses and purification workflows 19, ultimately leading to improved process performance, more reproducible control of HCP amounts and an ability to assess the ‘risk’ of HCPs, and assurance of product quality.

## Supporting information

As a service to our authors and readers, this journal provides supporting information supplied by the authors. Such materials are peer reviewed and may be re‐organized for online delivery, but are not copy‐edited or typeset. Technical support issues arising from supporting information (other than missing files) should be addressed to the authors.

Supporting Information
