# Supplementary material for: Quantitative definition and monitoring of the host cell protein proteome using iTRAQ – a study of an industrial mAb producing CHO‐S cell line
Source: Biotechnol J. 2016 Jun 22;11(8):1014–24. doi: 10.1002/biot.201500550 (PMC5031201; doi:10.1002/biot.201500550)
Supplement: Supplementary file 1 — Supporting Information [file BIOT-11-1014-s001.pdf]

Biotechnology
Journal

Supporting Information for DOI 10.1002/biot.201500550

Quantitative definition and monitoring of the host cell protein proteome using iTRAQ – a study of an industrial mAb producing CHO-S cell line

Lesley M. Chiverton, Caroline Evans, Jagroop Pandhal, Andrew R. Landels, Byron J. Rees, Peter R. Levison, Phillip C. Wright, C. Mark Smales

© 2016 Wiley-VCH Verlag GmbH & Co. KGaA, Weinheim

Supplementary File S1

Sample preparation for iTRAQ proteomic analysis, HILIC-HPLC fractionation of iTRAQ labelled peptides, mass spectrometry analysis, proteomic data identification, and proteomic data analysis

Sample preparation for iTRAQ proteomic analysis

The total protein concentration in the HCCF, post-Protein A eluate and flow through, post-cation eluate and post-anion flow through were determined using the method of Bradford [30] and, subsequently, 75 µg of total protein from each sample was processed for iTRAQ analysis. Samples were buffer exchanged using commercially available Vivaspin® columns (Sartorius UK, 5 kDa cut off) into triethylammonium bicarbonate, pH 8.5 (Sigma-Aldrich, Poole, Dorset), reduced, alkylated, and tryptically digested before being labelled with the appropriate iTRAQ label, according to manufacturer's instructions (ABSciex, Warrington, UK). Due to the cost and size of the 100 L wavebag experiment, technical sample replicates were analysed. Samples for each iTRAQ experiment were combined (experiment 1 – two replicates of HCCF, Protein A flow through, Protein A eluate; experiment 2 – two replicates of HCCF, Protein A eluate, cation exchange eluate and anion exchange flow through) then vacuum concentrated and fractionated by offline hydrophilic interaction liquid chromatography (HILIC)-HPLC, as described below.

HILIC-HPLC fractionation of iTRAQ labelled peptides

Peptide fractionation was performed using a 200 mm PolyHYDROXYETHYL-A (5 µm, 4.6 mm ID, 200 Å, PolyLC) analytical HILIC column (Columbia, MS, USA) using an Agilent 1100-series HPLC (Agilent, Berkshire UK) or U3000 HPLC system

(Thermo, Hemel Hempstead, UK). Samples were loaded, using a 45 min isocratic transfer flow-through at 500 µL/min, to remove non/low interaction analytes (HILIC transfer buffer A: 80% MeCN, 10 mM ammonium formate, pH 3.0). Enriched hydrophilic bound samples were eluted with elution buffer B (HILIC: 5% MeCN, 10 mM ammonium formate, pH 5.0; SCX: 25% MeCN, 0.5 M KCl, 0.1% formic acid), using a 3 step linear gradient at 0–20% B (5 min), 20–60% B (50 min) and 60–100% B (10 min), at 500 µL/min with samples collected at 1 min intervals. The column was isocratically eluted for a further 10 min in 100% B and, finally, re-equilibrated in 100% transfer buffer A. Chromatography was monitored *in situ* at 280 nm.

Mass Spectrometry Analysis

Individual HILIC fractions were injected into a nano-LC-ESI-MS/MS system for analysis. Mass spectrometry was performed using a Q-Star XL Hybrid ESI Quadrupole time-of flight tandem mass spectrometer, ESI-qQ-TOF-MS/MS (ABSciex, Warrington, UK), coupled to an online capillary liquid chromatography system (U3000 split loop system, Dionex/Thermo, UK). The peptide mixture was separated on a PepMap C-18 RP capillary column (Thermo, Hemel Hempstead, UK), with a constant flow rate of 0.3 µl/min. All samples were desalted online using a 5 cm, 300 µm i.d. C_{18} PepMap trap cartridge using 0.1% TFA and 3% ACN for 15 min, and eluted to a 15 cm, 75 µm i.d., 3 micron pore size LC-Packings C18 PepMap analytical column. The flow rate was 0.3 µl/min. The amount of peptide loaded onto the column correspond to 500 ng – 1 µg, injected in a volume of 6 µl. The LC gradient started with 3% Buffer B (0.1% formic acid in 97% acetonitrile) and 97% Buffer A (0.1% formic acid in 3% acetonitrile) for 3 min, followed by gradient of 3–30% Buffer B over 90 min, then 90% Buffer B for 7 min, and, finally, 3% Buffer B

for 8 min. The mass spectrometer was set to perform data acquisition in the positive ion mode, with a selected mass range of 300–2,000 m/z. Peptides with +2 to +4 charge states were selected for tandem mass spectrometry, and the time of summation of MS/MS events was set to 3 sec. The two most abundantly charged peptides above a five count threshold were selected for MS/MS, and dynamically excluded for 60 sec. Two injections were performed for each HILIC fraction analysed (40 for 6 sample iTRAQ, 35 for 8 sample iTRAQ).

Proteomic data identification

Identification of peptide precursor and fragments was performed by database searching against the Uniprot Chinese Hamster Ovary database (downloaded Jan 2015, 23884 entries) to which the sequence of the recombinant mAb (heavy and light chains) had been added. Parameters for searching using MASCOT software (version 2.3, Matrix Science, London, UK) were set as follows: MS tolerance and MS/MS tolerances were set at 1.2 and 0.6 Da respectively, charge state +2, +3 and +4. Target database search space was restricted to tryptic peptides with a maximum of 1 missed cleavage. Modifications were set as: 8-plex iTRAQ mass shifts (fixed, +304 Da, K and N-term), methylthiol (fixed, +46 Da) and oxidation of methionine (variable, +16 Da). Reporter ion intensities were retrieved for all matched spectra and post-processing was carried out using Mathematica 10 software (Wolfram, Long Hanborough, UK). A false discovery rate (FDR) of 1% was set, using values determined by searching a reverse decoy database [31]. The list of confidently identified spectra were normalised using median correction, to account for technical variation between samples, then filtered to exclude PSMs from proteins with fewer than 3 unique peptides.

Proteomic data analysis

The proteomic analysis was undertaken in two parts: the first determined if a protein was present at a detectable level following each purification step, and the second looked quantitatively at how the levels of proteins varied between the different stages of purification. These two steps were carried out separately and then merged together to produce the final dataset. To determine protein presence/absence at the detectable level, two labels in the first iTRAQ experiment (113 and 121) were kept unused, to provide an experimental dataset to model noise in the system and establish a limit of detection. The mean value of all the background noise was used as a cut-off and any iTRAQ reporter ion intensities below this point were converted to 0 for the purpose of this part of the analysis. For each stage of purification, peptide spectral matches (PSMs) lacking relative quantifications for each replicate were removed from analysis. Any protein, with 3 or more unique peptides remaining after this were considered to be detectable at that purification stage.

To investigate changing protein amounts throughout purification, a value equal to 1% of the mean reporter ion intensity was added to all label quantifications for each PSM [32] to account for absent reporter ions intensities, which may have significance in terms of protein amount. The proteins were then quantified by taking the mean of the log-transformed PSMs, and these data were used to produce heatmap figures investigating global effects, using the gplots package in R. A consistent drop/rise in the amount of protein at any stage of purification was determined if the following equations were evaluated as true:

Decrease: $Max(A_i) - 0.9 \times Min(B_i) < 0$

Increase: $0.9 \times Min(A_i) - Max(B_i) > 0$

Where **A** and **B** are protein quants for the prior and posterior conditions respectively, and $_i$ indicates replicates. Both lists were then merged together, generating a list of proteins that were either consistently reduced or dropped below (our defined) limit of detection; as well as a list of proteins that were still detectable. Proteins that appeared to have increased in concentration relative to the previous stage were classed as preferentially retained.

Proteins were assigned as enriched or depleted during the process, using the following calculation based on analysis of mean iTRAQ ratio and taking replicates into account.

$\text{Max } [A_1,A_2] < 0.9 \text{ Min } [B_1,B_2]$ - Enriched in B relative to A

$0.9 \text{ Min } [A_1,A_2] > 0.9 \text{ Max } [B_1,B_2]$ – Depleted in B relative to A

Where A and B represent different sample types e.g. HCCF (A) or Protein A eluate (B). A 10% minimal clearance margin was included for stringency. The lists of proteins depleted and enriched, based on these criteria, are shown in Supplementary Table S3.

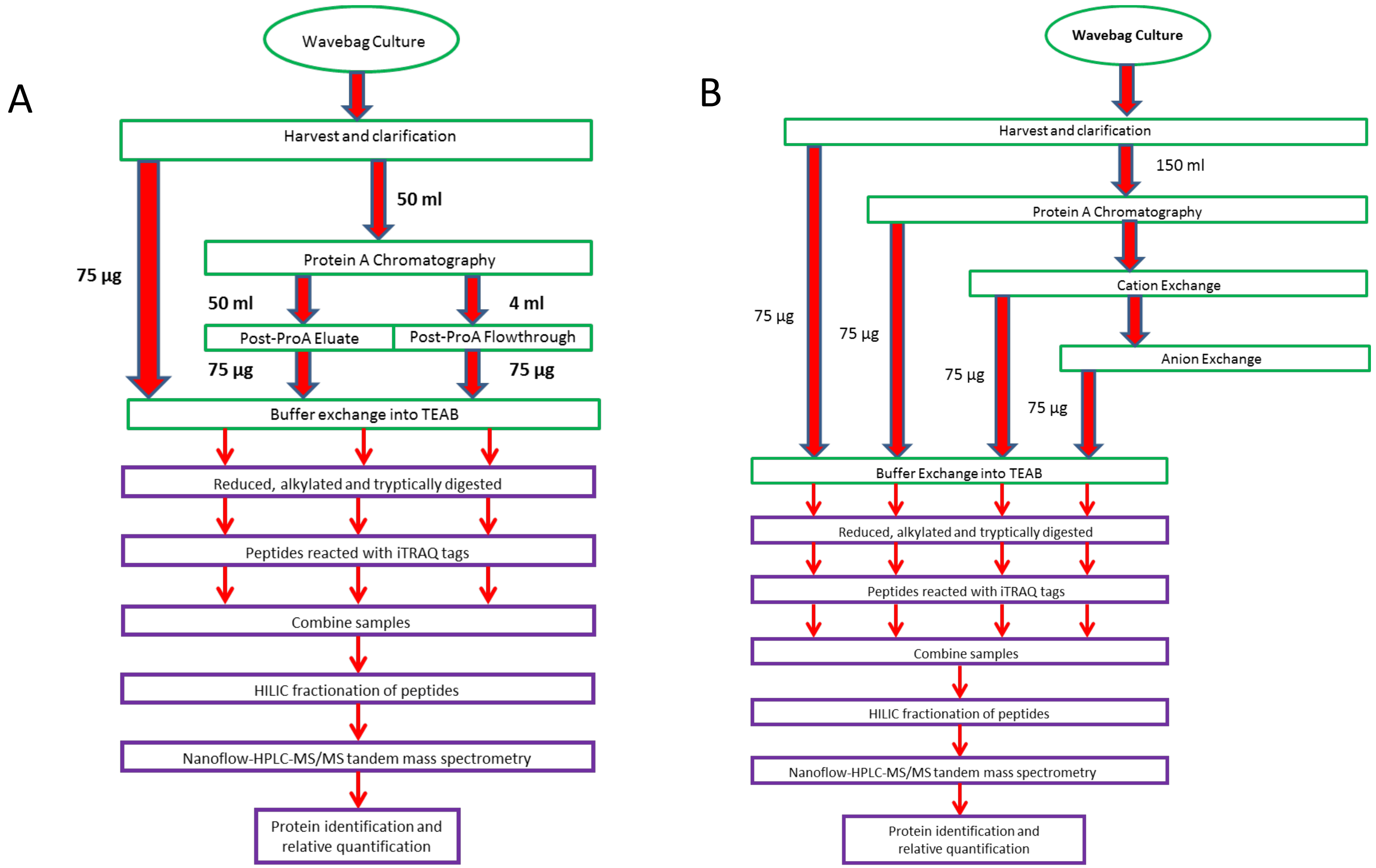

Supplementary Figure 1. Schematic representation of the experimental design for quantitative proteomics analysis of HCCF, post-Protein A flow through and eluate. A. iTRAQ labelling of duplicate samples of HCCF, post-Protein A flow through and eluate was performed followed by 2D LC peptide fractionation coupled to tandem mass spectrometry (LCMS/MS). B. iTRAQ labelling of duplicate samples of HCCF, post-Protein A eluate, post-cation exchange eluate and anion exchange flow through was performed. 2D chromatography coupled with tandem mass spectrometry (LC-MS/MS)-based was applied for protein identification and relative quantification.

Database	AC	# Peptides	# PSMs	Score	% Coverage
Uniprot:(Cricetulus barabensis griseus)[10029]	G3GR64	6	9	333.82	0.07228916
Uniprot:(Cricetulus barabensis griseus)[10029]	G3GR73	4	11	388.41	0.09213483
Uniprot:(Cricetulus barabensis griseus)[10029]	G3GR90	1	2	55.6	0.04210526
Uniprot:(Cricetulus barabensis griseus)[10029]	G3GRK2	1	1	22.15	0.00687285
Uniprot:(Cricetulus barabensis griseus)[10029]	G3GRP9	2	8	183.14	0.0278834
Uniprot:(Cricetulus barabensis griseus)[10029]	G3GRQ1	1	1	27.43	0.01827676
Uniprot:(Cricetulus barabensis griseus)[10029]	G3GRR9	1	1	20.43	0.01792574
Uniprot:(Cricetulus barabensis griseus)[10029]	G3GRX4	2	3	81.35	0.11267606
Uniprot:(Cricetulus barabensis griseus)[10029]	G3GRY5	1	1	21.66	0.03989362
Uniprot:(Cricetulus barabensis griseus)[10029]	G3GRY7	1	2	49.39	0.0859375
Uniprot:(Cricetulus barabensis griseus)[10029]	G3GS52	1	2	42.41	0.1
Uniprot:(Cricetulus barabensis griseus)[10029]	G3GS70	4	6	196.38	0.07615481
Uniprot:(Cricetulus barabensis griseus)[10029]	G3GSC8	1	3	74.03	0.01080432
Uniprot:(Cricetulus barabensis griseus)[10029]	G3GSE2	1	2	47.71	0.01115242
Uniprot:(Cricetulus barabensis griseus)[10029]	G3GSG4	4	6	146.94	0.13333333
Uniprot:(Cricetulus barabensis griseus)[10029]	G3GSJ7	1	1	22.12	0.02915452
Uniprot:(Cricetulus barabensis griseus)[10029]	G3GSK4	1	1	25.61	0.01908397
Uniprot:(Cricetulus barabensis griseus)[10029]	G3GSU4	1	7	327.52	0.0931677
Uniprot:(Cricetulus barabensis griseus)[10029]	G3GSX6	1	1	24.45	0.025
Uniprot:(Cricetulus barabensis griseus)[10029]	G3GT05	2	2	49.68	0.08780488
Uniprot:(Cricetulus barabensis griseus)[10029]	G3GT56	1	2	59.65	0.00891089
Uniprot:(Cricetulus barabensis griseus)[10029]	G3GT63	1	2	42.03	0.24615385
Uniprot:(Cricetulus barabensis griseus)[10029]	G3GTC7	4	9	309.96	0.20274914
Uniprot:(Cricetulus barabensis griseus)[10029]	G3GTC9	1	2	98.46	0.02767528
Uniprot:(Cricetulus barabensis griseus)[10029]	G3GTD9	1	1	22.73	0.00258695
Uniprot:(Cricetulus barabensis griseus)[10029]	G3GTK2	1	1	34.99	0.08035714
Uniprot:(Cricetulus barabensis griseus)[10029]	G3GTT2	4	49	1475.1	0.22377622
Uniprot:(Cricetulus barabensis griseus)[10029]	G3GTU9	1	1	20.34	0.01244344
Uniprot:(Cricetulus barabensis griseus)[10029]	G3GTV4	1	1	22.28	0.01062216
Uniprot:(Cricetulus barabensis griseus)[10029]	G3GTX5	5	9	296.9	0.08064516
Uniprot:(Cricetulus barabensis griseus)[10029]	G3GU60	4	23	853.58	0.22994652
Uniprot:(Cricetulus barabensis griseus)[10029]	G3GU87	1	1	20.48	0.01336898
Uniprot:(Cricetulus barabensis griseus)[10029]	G3GUM6	1	2	92.83	0.08076923
Uniprot:(Cricetulus barabensis griseus)[10029]	G3GUR0	1	1	23.38	0.01151079
Uniprot:(Cricetulus barabensis griseus)[10029]	G3GUR1	1	1	22.63	0.01134752
Uniprot:(Cricetulus barabensis griseus)[10029]	G3GUU5	14	32	1185.3	0.31781701
Uniprot:(Cricetulus barabensis griseus)[10029]	G3GUV3	1	1	23.27	0.0237069
Uniprot:(Cricetulus barabensis griseus)[10029]	G3GUV4	9	28	892.37	0.09006211
Uniprot:(Cricetulus barabensis griseus)[10029]	G3GV01	1	2	76.55	0.01624549
Uniprot:(Cricetulus barabensis griseus)[10029]	G3GV45	1	4	90.87	0.00736067
Uniprot:(Cricetulus barabensis griseus)[10029]	G3GVB5	2	3	68.48	0.14864865
Uniprot:(Cricetulus barabensis griseus)[10029]	G3GVD0	3	7	316.03	0.13066667
Uniprot:(Cricetulus barabensis griseus)[10029]	G3GVG4	1	1	20.58	0.0120614
Uniprot:(Cricetulus barabensis griseus)[10029]	G3GVM9	1	1	31.12	0.10810811

Uniprot:(Cricetulus barabensis griseus)[10029]	G3GVX1	5	5	121.27	0.024888
Uniprot:(Cricetulus barabensis griseus)[10029]	G3GVX2	3	8	316.39	0.21818182
Uniprot:(Cricetulus barabensis griseus)[10029]	G3GW01	1	1	20.05	0.00482426
Uniprot:(Cricetulus barabensis griseus)[10029]	G3GW53	1	4	123.36	0.00958467
Uniprot:(Cricetulus barabensis griseus)[10029]	G3GWB3	5	7	247.12	0.13675214
Uniprot:(Cricetulus barabensis griseus)[10029]	G3GWB5	1	1	22.12	0.02329749
Uniprot:(Cricetulus barabensis griseus)[10029]	G3GWB9	1	1	22.52	0.02
Uniprot:(Cricetulus barabensis griseus)[10029]	G3GWC2	1	2	43.12	0.0123119
Uniprot:(Cricetulus barabensis griseus)[10029]	G3GWC4	1	1	29.97	0.04059041
Uniprot:(Cricetulus barabensis griseus)[10029]	G3GWE9	1	1	32.13	0.01006036
Uniprot:(Cricetulus barabensis griseus)[10029]	G3GWF4	1	1	21.05	0.00771208
Uniprot:(Cricetulus barabensis griseus)[10029]	G3GWQ1	20	36	1478.8	0.22134733
Uniprot:(Cricetulus barabensis griseus)[10029]	G3GWR8	5	17	592.69	0.28773585
Uniprot:(Cricetulus barabensis griseus)[10029]	G3GWX6	1	1	32.05	0.02638522
Uniprot:(Cricetulus barabensis griseus)[10029]	G3GX09	1	1	24.41	0.01224944
Uniprot:(Cricetulus barabensis griseus)[10029]	G3GX21	1	1	20.55	0.02848101
Uniprot:(Cricetulus barabensis griseus)[10029]	G3GX38	1	2	79.45	0.12359551
Uniprot:(Cricetulus barabensis griseus)[10029]	G3GX96	2	4	125.32	0.11309524
Uniprot:(Cricetulus barabensis griseus)[10029]	G3GXA9	5	17	502.57	0.24456522
Uniprot:(Cricetulus barabensis griseus)[10029]	G3GXB0	4	7	270.86	0.2254902
Uniprot:(Cricetulus barabensis griseus)[10029]	G3GXD7	12	19	719.55	0.05833
Uniprot:(Cricetulus barabensis griseus)[10029]	G3GXS2	4	5	164.65	0.03933137
Uniprot:(Cricetulus barabensis griseus)[10029]	G3GXS3	1	1	27.71	0.02318841
Uniprot:(Cricetulus barabensis griseus)[10029]	G3GXV8	1	1	27.14	0.05232558
Uniprot:(Cricetulus barabensis griseus)[10029]	G3GXW5	1	1	21.27	0.01603774
Uniprot:(Cricetulus barabensis griseus)[10029]	G3GXW7	1	1	22.85	0.00632578
Uniprot:(Cricetulus barabensis griseus)[10029]	G3GXW9	1	2	60.99	0.01492537
Uniprot:(Cricetulus barabensis griseus)[10029]	G3GXX0	1	1	20.48	0.06976744
Uniprot:(Cricetulus barabensis griseus)[10029]	G3GXZ0	11	46	1762.6	0.18804665
Uniprot:(Cricetulus barabensis griseus)[10029]	G3GXZ8	1	1	20.38	0.04
Uniprot:(Cricetulus barabensis griseus)[10029]	G3GY12	1	1	24.15	0.00923361
Uniprot:(Cricetulus barabensis griseus)[10029]	G3GY17	4	4	119.69	0.03648425
Uniprot:(Cricetulus barabensis griseus)[10029]	G3GY52	1	1	20.24	0.00672646
Uniprot:(Cricetulus barabensis griseus)[10029]	G3GY55	1	1	20.01	0.01405152
Uniprot:(Cricetulus barabensis griseus)[10029]	G3GY95	2	8	402.47	0.40789474
Uniprot:(Cricetulus barabensis griseus)[10029]	G3GYB7	1	1	30.58	0.09090909
Uniprot:(Cricetulus barabensis griseus)[10029]	G3GYD1	1	1	26.03	0.01380671
Uniprot:(Cricetulus barabensis griseus)[10029]	G3GYG0	2	3	145.31	0.1372549
Uniprot:(Cricetulus barabensis griseus)[10029]	G3GYP7	3	6	156.31	0.11728395
Uniprot:(Cricetulus barabensis griseus)[10029]	G3GYP9	7	49	1790	0.35678392
Uniprot:(Cricetulus barabensis griseus)[10029]	G3GYU3	1	1	24.51	0.0311804
Uniprot:(Cricetulus barabensis griseus)[10029]	G3GYY2	1	2	63.63	0.01676829
Uniprot:(Cricetulus barabensis griseus)[10029]	G3GYY6	2	4	150.88	0.04864865
Uniprot:(Cricetulus barabensis griseus)[10029]	G3GYZ1	2	13	460.19	0.04259635
Uniprot:(Cricetulus barabensis griseus)[10029]	G3GZ00	1	1	21.49	0.06060606

Uniprot:(Cricetulus barabensis griseus)[10029]	G3GZ81	1	3	96.78	0.05780347
Uniprot:(Cricetulus barabensis griseus)[10029]	G3GZ90	3	6	181.12	0.04718218
Uniprot:(Cricetulus barabensis griseus)[10029]	G3GZ94	1	2	78.59	0.00337838
Uniprot:(Cricetulus barabensis griseus)[10029]	G3GZA9	1	1	20.36	0.06504065
Uniprot:(Cricetulus barabensis griseus)[10029]	G3GZB2	13	19	502.56	0.34177215
Uniprot:(Cricetulus barabensis griseus)[10029]	G3GZC9	1	1	20.32	0.00744879
Uniprot:(Cricetulus barabensis griseus)[10029]	G3GZD2	5	16	454.74	0.13986014
Uniprot:(Cricetulus barabensis griseus)[10029]	G3GZE2	1	1	20.43	0.00393959
Uniprot:(Cricetulus barabensis griseus)[10029]	G3GZE6	1	4	177.44	0.0215311
Uniprot:(Cricetulus barabensis griseus)[10029]	G3GZF1	1	1	21.39	0.00881705
Uniprot:(Cricetulus barabensis griseus)[10029]	G3GZF3	1	1	20.4	0.01351351
Uniprot:(Cricetulus barabensis griseus)[10029]	G3GZG4	1	2	86.9	0.08571429
Uniprot:(Cricetulus barabensis griseus)[10029]	G3GZL7	1	3	70.57	0.03804348
Uniprot:(Cricetulus barabensis griseus)[10029]	G3GZP6	1	1	26.97	0.01333333
Uniprot:(Cricetulus barabensis griseus)[10029]	G3GZR1	1	1	21.59	0.01043841
Uniprot:(Cricetulus barabensis griseus)[10029]	G3GZW8	3	27	943.16	0.34831461
Uniprot:(Cricetulus barabensis griseus)[10029]	G3GZZ0	4	10	374.47	0.14043584
Uniprot:(Cricetulus barabensis griseus)[10029]	G3H066	1	4	145.11	0.02917342
Uniprot:(Cricetulus barabensis griseus)[10029]	G3H0B6	1	1	26.37	0.00904977
Uniprot:(Cricetulus barabensis griseus)[10029]	G3H0C2	3	4	103.49	0.08812261
Uniprot:(Cricetulus barabensis griseus)[10029]	G3H0C9	3	5	169.86	0.21794872
Uniprot:(Cricetulus barabensis griseus)[10029]	G3H0E4	21	32	1269.8	0.12569953
Uniprot:(Cricetulus barabensis griseus)[10029]	G3H0L9	4	10	315.46	0.12389381
Uniprot:(Cricetulus barabensis griseus)[10029]	G3H0S4	5	7	270.98	0.1512605
Uniprot:(Cricetulus barabensis griseus)[10029]	G3H0S7	4	10	301.92	0.88888889
Uniprot:(Cricetulus barabensis griseus)[10029]	G3H0T6	1	1	20.31	0.02095238
Uniprot:(Cricetulus barabensis griseus)[10029]	G3H0U6	21	63	2053.2	0.54504505
Uniprot:(Cricetulus barabensis griseus)[10029]	G3H0U9	1	4	124.45	0.02398082
Uniprot:(Cricetulus barabensis griseus)[10029]	G3H0V1	1	3	97.36	0.00386847
Uniprot:(Cricetulus barabensis griseus)[10029]	G3H177	1	1	21.45	0.02808989
Uniprot:(Cricetulus barabensis griseus)[10029]	G3H194	1	1	34.29	0.05960265
Uniprot:(Cricetulus barabensis griseus)[10029]	G3H1A1	1	1	21.39	0.02887139
Uniprot:(Cricetulus barabensis griseus)[10029]	G3H1D5	1	1	22.78	0.01538462
Uniprot:(Cricetulus barabensis griseus)[10029]	G3H1G3	1	1	23.01	0.00758534
Uniprot:(Cricetulus barabensis griseus)[10029]	G3H1I2	1	1	24.92	0.06622517
Uniprot:(Cricetulus barabensis griseus)[10029]	G3H1K9	6	14	483.34	0.06644518
Uniprot:(Cricetulus barabensis griseus)[10029]	G3H1M4	1	1	32.39	0.02539683
Uniprot:(Cricetulus barabensis griseus)[10029]	G3H1Q2	1	1	20.4	0.01217656
Uniprot:(Cricetulus barabensis griseus)[10029]	G3H1U3	1	2	52.34	0.07407407
Uniprot:(Cricetulus barabensis griseus)[10029]	G3H1W1	2	2	49.29	0.01179392
Uniprot:(Cricetulus barabensis griseus)[10029]	G3H1W4	2	5	214.04	0.0344086
Uniprot:(Cricetulus barabensis griseus)[10029]	G3H1Z4	1	2	47.43	0.03557312
Uniprot:(Cricetulus barabensis griseus)[10029]	G3H235	1	2	41.29	0.04558405
Uniprot:(Cricetulus barabensis griseus)[10029]	G3H289	1	1	30.25	0.03080569
Uniprot:(Cricetulus barabensis griseus)[10029]	G3H295	1	1	22.86	0.01125176

Uniprot:(Cricetulus barabensis griseus)[10029]	G3H2C4	4	6	228.77	0.08944544
Uniprot:(Cricetulus barabensis griseus)[10029]	G3H2E2	1	3	86.07	0.05687204
Uniprot:(Cricetulus barabensis griseus)[10029]	G3H2J8	1	3	94.83	0.03546099
Uniprot:(Cricetulus barabensis griseus)[10029]	G3H2K2	3	12	398.06	0.34285714
Uniprot:(Cricetulus barabensis griseus)[10029]	G3H2P3	1	3	98.51	0.01538462
Uniprot:(Cricetulus barabensis griseus)[10029]	G3H2Q4	1	2	52.42	0.04897959
Uniprot:(Cricetulus barabensis griseus)[10029]	G3H2S9	1	1	20.23	0.02255639
Uniprot:(Cricetulus barabensis griseus)[10029]	G3H2T6	2	6	201.47	0.22330097
Uniprot:(Cricetulus barabensis griseus)[10029]	G3H2T8	2	10	387.22	0.21538462
Uniprot:(Cricetulus barabensis griseus)[10029]	G3H2W6	1	1	22.82	0.01243339
Uniprot:(Cricetulus barabensis griseus)[10029]	G3H2Z9	1	1	24.12	0.01453958
Uniprot:(Cricetulus barabensis griseus)[10029]	G3H303	1	1	21.41	0.03030303
Uniprot:(Cricetulus barabensis griseus)[10029]	G3H308	1	1	23.96	0.02051282
Uniprot:(Cricetulus barabensis griseus)[10029]	G3H319	2	3	86.69	0.20238095
Uniprot:(Cricetulus barabensis griseus)[10029]	G3H352	1	1	24.91	0.00130662
Uniprot:(Cricetulus barabensis griseus)[10029]	G3H354	11	34	1215.2	0.36144578
Uniprot:(Cricetulus barabensis griseus)[10029]	G3H3B9	1	1	20.32	0.05508475
Uniprot:(Cricetulus barabensis griseus)[10029]	G3H3C3	1	1	20.61	0.00811688
Uniprot:(Cricetulus barabensis griseus)[10029]	G3H3C6	1	1	30.5	0.03855422
Uniprot:(Cricetulus barabensis griseus)[10029]	G3H3D3	3	9	279.45	0.09324009
Uniprot:(Cricetulus barabensis griseus)[10029]	G3H3E4	3	10	363.82	0.07665505
Uniprot:(Cricetulus barabensis griseus)[10029]	G3H3E9	1	1	23.5	0.00155452
Uniprot:(Cricetulus barabensis griseus)[10029]	G3H3G2	1	2	42.9	0.1038961
Uniprot:(Cricetulus barabensis griseus)[10029]	G3H3J2	1	1	20.88	0.01263538
Uniprot:(Cricetulus barabensis griseus)[10029]	G3H3Q1	10	47	1912.6	0.33898305
Uniprot:(Cricetulus barabensis griseus)[10029]	G3H3X1	2	2	71.28	0.03594771
Uniprot:(Cricetulus barabensis griseus)[10029]	G3H497	1	1	34.25	0.02325581
Uniprot:(Cricetulus barabensis griseus)[10029]	G3H4A6	1	1	23.3	0.00275314
Uniprot:(Cricetulus barabensis griseus)[10029]	G3H4I2	6	20	710.37	0.19245283
Uniprot:(Cricetulus barabensis griseus)[10029]	G3H4K5	1	2	71.11	0.078125
Uniprot:(Cricetulus barabensis griseus)[10029]	G3H4S3	1	3	75.96	0.03341902
Uniprot:(Cricetulus barabensis griseus)[10029]	G3H4T5	5	8	279.42	0.08322663
Uniprot:(Cricetulus barabensis griseus)[10029]	G3H4V1	5	8	259.78	0.17445483
Uniprot:(Cricetulus barabensis griseus)[10029]	G3H4Y9	1	1	21.02	0.00775194
Uniprot:(Cricetulus barabensis griseus)[10029]	G3H4Z8	3	7	296.02	0.0615142
Uniprot:(Cricetulus barabensis griseus)[10029]	G3H533	7	21	678.92	0.375
Uniprot:(Cricetulus barabensis griseus)[10029]	G3H559	2	2	101.89	0.01910828
Uniprot:(Cricetulus barabensis griseus)[10029]	G3H577	4	13	337.49	0.16230367
Uniprot:(Cricetulus barabensis griseus)[10029]	G3H584	3	12	452.27	0.12851406
Uniprot:(Cricetulus barabensis griseus)[10029]	G3H5D5	1	3	151.55	0.13445378
Uniprot:(Cricetulus barabensis griseus)[10029]	G3H5V0	5	6	185.24	0.03484689
Uniprot:(Cricetulus barabensis griseus)[10029]	G3H5W0	4	10	477.47	0.30412371
Uniprot:(Cricetulus barabensis griseus)[10029]	G3H5W5	1	1	27.83	0.03964758
Uniprot:(Cricetulus barabensis griseus)[10029]	G3H5X3	1	2	51.77	0.00725163
Uniprot:(Cricetulus barabensis griseus)[10029]	G3H605	1	1	29.34	0.02227172

Uniprot:(Cricetulus barabensis griseus)[10029]	G3H612	1	1	21.9	0.11666667
Uniprot:(Cricetulus barabensis griseus)[10029]	G3H682	1	4	156.47	0.05555556
Uniprot:(Cricetulus barabensis griseus)[10029]	G3H697	6	7	269.18	0.1205036
Uniprot:(Cricetulus barabensis griseus)[10029]	G3H6C5	1	2	79.89	0.02902375
Uniprot:(Cricetulus barabensis griseus)[10029]	G3H6I5	3	8	204.06	0.0755287
Uniprot:(Cricetulus barabensis griseus)[10029]	G3H6J7	1	1	27.72	0.03732809
Uniprot:(Cricetulus barabensis griseus)[10029]	G3H6K1	1	1	23.44	0.04166667
Uniprot:(Cricetulus barabensis griseus)[10029]	G3H6M5	1	1	26.99	0.01972387
Uniprot:(Cricetulus barabensis griseus)[10029]	G3H6P3	1	1	21.38	0.01860465
Uniprot:(Cricetulus barabensis griseus)[10029]	G3H6R3	1	1	22	0.02888087
Uniprot:(Cricetulus barabensis griseus)[10029]	G3H6T5	5	19	630.65	0.05343512
Uniprot:(Cricetulus barabensis griseus)[10029]	G3H6Y5	1	2	103.03	0.17721519
Uniprot:(Cricetulus barabensis griseus)[10029]	G3H6Y6	1	2	90.46	0.03225807
Uniprot:(Cricetulus barabensis griseus)[10029]	G3H705	3	10	284.15	0.2745098
Uniprot:(Cricetulus barabensis griseus)[10029]	G3H748	1	1	29.18	0.00850159
Uniprot:(Cricetulus barabensis griseus)[10029]	G3H771	2	9	275.12	0.05053192
Uniprot:(Cricetulus barabensis griseus)[10029]	G3H773	1	1	24.01	0.01449275
Uniprot:(Cricetulus barabensis griseus)[10029]	G3H7B3	3	8	256.67	0.12455516
Uniprot:(Cricetulus barabensis griseus)[10029]	G3H7I2	1	1	20.6	0.00820152
Uniprot:(Cricetulus barabensis griseus)[10029]	G3H7I6	2	3	93.14	0.03365385
Uniprot:(Cricetulus barabensis griseus)[10029]	G3H7K5	2	4	234.3	0.0702403
Uniprot:(Cricetulus barabensis griseus)[10029]	G3H7N7	1	1	22.46	0.00436681
Uniprot:(Cricetulus barabensis griseus)[10029]	G3H7Q5	1	1	24.21	0.02877698
Uniprot:(Cricetulus barabensis griseus)[10029]	G3H7X7	1	1	20.12	0.00959693
Uniprot:(Cricetulus barabensis griseus)[10029]	G3H7Z2	5	6	252.44	0.34170854
Uniprot:(Cricetulus barabensis griseus)[10029]	G3H873	1	1	20.11	0.00985663
Uniprot:(Cricetulus barabensis griseus)[10029]	G3H892	1	1	20.47	0.01785714
Uniprot:(Cricetulus barabensis griseus)[10029]	G3H8A8	1	2	87.12	0.06912442
Uniprot:(Cricetulus barabensis griseus)[10029]	G3H8D9	1	1	23.67	0.01914894
Uniprot:(Cricetulus barabensis griseus)[10029]	G3H8E2	1	1	23.66	0.0264993
Uniprot:(Cricetulus barabensis griseus)[10029]	G3H8F4	3	3	91.62	0.04031355
Uniprot:(Cricetulus barabensis griseus)[10029]	G3H8G3	1	1	23.39	0.07518797
Uniprot:(Cricetulus barabensis griseus)[10029]	G3H8G9	1	5	110.53	0.00680851
Uniprot:(Cricetulus barabensis griseus)[10029]	G3H8K2	1	2	68.11	0.02439024
Uniprot:(Cricetulus barabensis griseus)[10029]	G3H8N1	1	2	81.14	0.01949541
Uniprot:(Cricetulus barabensis griseus)[10029]	G3H8Q0	1	4	150.42	0.04761905
Uniprot:(Cricetulus barabensis griseus)[10029]	G3H8T0	1	1	21.21	0.01028571
Uniprot:(Cricetulus barabensis griseus)[10029]	G3H8V1	6	11	404.49	0.08169014
Uniprot:(Cricetulus barabensis griseus)[10029]	G3H8V4	6	26	874.78	0.13590264
Uniprot:(Cricetulus barabensis griseus)[10029]	G3H8V5	6	32	1034.7	0.16210526
Uniprot:(Cricetulus barabensis griseus)[10029]	G3H8Y4	3	5	135.6	0.04521964
Uniprot:(Cricetulus barabensis griseus)[10029]	G3H8Y5	3	7	234.66	0.0486618
Uniprot:(Cricetulus barabensis griseus)[10029]	G3H919	1	1	22.06	0.00198469
Uniprot:(Cricetulus barabensis griseus)[10029]	G3H928	3	10	355.66	0.24647887
Uniprot:(Cricetulus barabensis griseus)[10029]	G3H932	1	4	118.07	0.06735751

Uniprot:(Cricetulus barabensis griseus)[10029]	G3H935	5	10	359.91	0.09280303
Uniprot:(Cricetulus barabensis griseus)[10029]	G3H992	1	1	20.83	0.08695652
Uniprot:(Cricetulus barabensis griseus)[10029]	G3H9F7	1	1	27.17	0.01816347
Uniprot:(Cricetulus barabensis griseus)[10029]	G3H9H7	1	1	21.09	0.02061856
Uniprot:(Cricetulus barabensis griseus)[10029]	G3H9J2	1	1	21.59	0.03738318
Uniprot:(Cricetulus barabensis griseus)[10029]	G3H9L8	1	1	20.28	0.20779221
Uniprot:(Cricetulus barabensis griseus)[10029]	G3H9V0	2	3	90.41	0.07960199
Uniprot:(Cricetulus barabensis griseus)[10029]	G3HA23	1	2	51.14	0.03690037
Uniprot:(Cricetulus barabensis griseus)[10029]	G3HA54	5	18	610.33	0.19259259
Uniprot:(Cricetulus barabensis griseus)[10029]	G3HA55	2	2	46.63	0.2962963
Uniprot:(Cricetulus barabensis griseus)[10029]	G3HAI3	1	3	76	0.01372213
Uniprot:(Cricetulus barabensis griseus)[10029]	G3HAI8	1	1	20.51	0.00513023
Uniprot:(Cricetulus barabensis griseus)[10029]	G3HAJ0	1	1	24.41	0.00263591
Uniprot:(Cricetulus barabensis griseus)[10029]	G3HAJ1	1	2	83.57	0.09322034
Uniprot:(Cricetulus barabensis griseus)[10029]	G3HAL2	1	1	30.66	0.00817439
Uniprot:(Cricetulus barabensis griseus)[10029]	G3HAM4	1	1	20.76	0.01347709
Uniprot:(Cricetulus barabensis griseus)[10029]	G3HAN8	2	2	65.53	0.04861111
Uniprot:(Cricetulus barabensis griseus)[10029]	G3HAP1	1	3	73.08	0.02517986
Uniprot:(Cricetulus barabensis griseus)[10029]	G3HAP7	6	17	612.12	0.13924051
Uniprot:(Cricetulus barabensis griseus)[10029]	G3HAX3	1	1	28	0.00692841
Uniprot:(Cricetulus barabensis griseus)[10029]	G3HB04	8	31	1671.8	0.42084942
Uniprot:(Cricetulus barabensis griseus)[10029]	G3HB78	1	1	25.38	0.02325581
Uniprot:(Cricetulus barabensis griseus)[10029]	G3HBD3	4	15	429.23	0.30921053
Uniprot:(Cricetulus barabensis griseus)[10029]	G3HBD4	4	14	436.16	0.35526316
Uniprot:(Cricetulus barabensis griseus)[10029]	G3HBG7	1	1	25.65	0.00758294
Uniprot:(Cricetulus barabensis griseus)[10029]	G3HBG8	1	3	144.39	0.13664596
Uniprot:(Cricetulus barabensis griseus)[10029]	G3HBI9	3	5	138.3	0.05671078
Uniprot:(Cricetulus barabensis griseus)[10029]	G3HBS5	1	1	21.04	0.01158749
Uniprot:(Cricetulus barabensis griseus)[10029]	G3HBU0	1	1	24.5	0.01467505
Uniprot:(Cricetulus barabensis griseus)[10029]	G3HC01	1	1	62.25	0.03934426
Uniprot:(Cricetulus barabensis griseus)[10029]	G3HC13	1	1	21.68	0.01345292
Uniprot:(Cricetulus barabensis griseus)[10029]	G3HC14	1	1	25	0.0259366
Uniprot:(Cricetulus barabensis griseus)[10029]	G3HC20	1	2	44.54	0.02409639
Uniprot:(Cricetulus barabensis griseus)[10029]	G3HC25	4	13	459.36	0.3877551
Uniprot:(Cricetulus barabensis griseus)[10029]	G3HC29	3	15	410.17	0.23762376
Uniprot:(Cricetulus barabensis griseus)[10029]	G3HC30	2	6	215.37	0.24731183
Uniprot:(Cricetulus barabensis griseus)[10029]	G3HC31	4	20	693.6	0.5505618
Uniprot:(Cricetulus barabensis griseus)[10029]	G3HC39	1	4	115.92	0.024
Uniprot:(Cricetulus barabensis griseus)[10029]	G3HC84	5	5	121.08	0.09223301
Uniprot:(Cricetulus barabensis griseus)[10029]	G3HCD5	1	1	20.76	0.00537634
Uniprot:(Cricetulus barabensis griseus)[10029]	G3HCL3	1	4	210.03	0.04885994
Uniprot:(Cricetulus barabensis griseus)[10029]	G3HCL6	1	2	44.68	0.00369004
Uniprot:(Cricetulus barabensis griseus)[10029]	G3HCL9	1	1	24.3	0.06463878
Uniprot:(Cricetulus barabensis griseus)[10029]	G3HCP7	1	1	21.11	0.03629032
Uniprot:(Cricetulus barabensis griseus)[10029]	G3HCQ3	1	1	23.3	0.0295203

Uniprot:(Cricetulus barabensis griseus)[10029]	G3HCW9	3	7	308.65	0.18181818
Uniprot:(Cricetulus barabensis griseus)[10029]	G3HCX3	2	3	68.2	0.04043127
Uniprot:(Cricetulus barabensis griseus)[10029]	G3HCX8	9	24	812.37	0.23261391
Uniprot:(Cricetulus barabensis griseus)[10029]	G3HD20	1	1	22.42	0.02102102
Uniprot:(Cricetulus barabensis griseus)[10029]	G3HD97	4	5	160.74	0.12470024
Uniprot:(Cricetulus barabensis griseus)[10029]	G3HDA5	1	9	260.47	0.06306306
Uniprot:(Cricetulus barabensis griseus)[10029]	G3HDE3	1	1	47.32	0.09623431
Uniprot:(Cricetulus barabensis griseus)[10029]	G3HDE5	1	3	108.86	0.05116279
Uniprot:(Cricetulus barabensis griseus)[10029]	G3HDL6	2	3	81.28	0.08888889
Uniprot:(Cricetulus barabensis griseus)[10029]	G3HDN1	1	1	23.43	0.02329451
Uniprot:(Cricetulus barabensis griseus)[10029]	G3HDQ1	4	7	188.65	0.11811024
Uniprot:(Cricetulus barabensis griseus)[10029]	G3HDQ2	5	10	247.07	0.14371258
Uniprot:(Cricetulus barabensis griseus)[10029]	G3HDR3	2	2	52.85	0.05289673
Uniprot:(Cricetulus barabensis griseus)[10029]	G3HDS5	2	7	171.94	0.10294118
Uniprot:(Cricetulus barabensis griseus)[10029]	G3HDT6	3	5	181.23	0.09667674
Uniprot:(Cricetulus barabensis griseus)[10029]	G3HDT7	1	1	36.21	0.07142857
Uniprot:(Cricetulus barabensis griseus)[10029]	G3HE06	1	1	20.24	0.14
Uniprot:(Cricetulus barabensis griseus)[10029]	G3HE84	1	1	22.97	0.02985075
Uniprot:(Cricetulus barabensis griseus)[10029]	G3HEA3	1	1	20.09	0.05537459
Uniprot:(Cricetulus barabensis griseus)[10029]	G3HEH9	1	1	29.93	0.01727447
Uniprot:(Cricetulus barabensis griseus)[10029]	G3HEI4	1	1	24.95	0.01851852
Uniprot:(Cricetulus barabensis griseus)[10029]	G3HEJ4	1	1	30.21	0.00854093
Uniprot:(Cricetulus barabensis griseus)[10029]	G3HEK6	1	1	20.9	0.01470588
Uniprot:(Cricetulus barabensis griseus)[10029]	G3HEL2	2	4	114.29	0.08097166
Uniprot:(Cricetulus barabensis griseus)[10029]	G3HEQ3	1	2	90.36	0.05
Uniprot:(Cricetulus barabensis griseus)[10029]	G3HET8	1	3	88.79	0.06666667
Uniprot:(Cricetulus barabensis griseus)[10029]	G3HEV1	1	1	24.16	0.01601831
Uniprot:(Cricetulus barabensis griseus)[10029]	G3HEW0	1	2	50.78	0.16161616
Uniprot:(Cricetulus barabensis griseus)[10029]	G3HEZ0	1	1	32.73	0.01983471
Uniprot:(Cricetulus barabensis griseus)[10029]	G3HEZ1	2	2	72.36	0.06930693
Uniprot:(Cricetulus barabensis griseus)[10029]	G3HF16	1	1	25.7	0.01470588
Uniprot:(Cricetulus barabensis griseus)[10029]	G3HF18	1	2	72.23	0.05714286
Uniprot:(Cricetulus barabensis griseus)[10029]	G3HF33	1	1	22.75	0.07317073
Uniprot:(Cricetulus barabensis griseus)[10029]	G3HF47	1	1	20.82	0.02672293
Uniprot:(Cricetulus barabensis griseus)[10029]	G3HF75	1	1	23.79	0.05660377
Uniprot:(Cricetulus barabensis griseus)[10029]	G3HFG6	1	2	47.77	0.00435256
Uniprot:(Cricetulus barabensis griseus)[10029]	G3HFG7	2	4	145.7	0.15882353
Uniprot:(Cricetulus barabensis griseus)[10029]	G3HFI4	1	2	42.78	0.02786378
Uniprot:(Cricetulus barabensis griseus)[10029]	G3HFJ1	1	2	57.98	0.03244838
Uniprot:(Cricetulus barabensis griseus)[10029]	G3HFM2	1	1	20.24	0.14893617
Uniprot:(Cricetulus barabensis griseus)[10029]	G3HFM4	12	18	596.42	0.05995388
Uniprot:(Cricetulus barabensis griseus)[10029]	G3HFP1	1	2	116.6	0.02572899
Uniprot:(Cricetulus barabensis griseus)[10029]	G3HFQ2	2	3	69.46	0.0097561
Uniprot:(Cricetulus barabensis griseus)[10029]	G3HFR8	1	1	21.01	0.06666667
Uniprot:(Cricetulus barabensis griseus)[10029]	G3HFU9	1	1	24.42	0.04109589

Uniprot:(Cricetulus barabensis griseus)[10029]	G3HFW4	1	1	21.28	0.01570681
Uniprot:(Cricetulus barabensis griseus)[10029]	G3HFY1	2	3	87.39	0.05133929
Uniprot:(Cricetulus barabensis griseus)[10029]	G3HFY9	2	3	112.49	0.22077922
Uniprot:(Cricetulus barabensis griseus)[10029]	G3HG69	1	2	63.66	0.04390244
Uniprot:(Cricetulus barabensis griseus)[10029]	G3HG71	1	1	21.07	0.07407407
Uniprot:(Cricetulus barabensis griseus)[10029]	G3HG83	5	11	308.15	0.09357798
Uniprot:(Cricetulus barabensis griseus)[10029]	G3HG95	12	28	971.83	0.20603908
Uniprot:(Cricetulus barabensis griseus)[10029]	G3HGI4	1	2	53.27	0.09210526
Uniprot:(Cricetulus barabensis griseus)[10029]	G3HGJ5	1	1	23.83	0.05963303
Uniprot:(Cricetulus barabensis griseus)[10029]	G3HGJ9	1	17	369.53	0.03236246
Uniprot:(Cricetulus barabensis griseus)[10029]	G3HGK3	2	15	451.09	0.15178571
Uniprot:(Cricetulus barabensis griseus)[10029]	G3HGM2	1	1	20.19	0.06493507
Uniprot:(Cricetulus barabensis griseus)[10029]	G3HGM6	4	9	524.14	0.15028902
Uniprot:(Cricetulus barabensis griseus)[10029]	G3HGP4	3	4	132.72	0.09319899
Uniprot:(Cricetulus barabensis griseus)[10029]	G3HGP6	2	3	75.01	0.03597122
Uniprot:(Cricetulus barabensis griseus)[10029]	G3HGQ6	1	2	59.76	0.01004304
Uniprot:(Cricetulus barabensis griseus)[10029]	G3HGW5	1	2	52.89	0.01965602
Uniprot:(Cricetulus barabensis griseus)[10029]	G3HGY8	6	13	482.99	0.1187905
Uniprot:(Cricetulus barabensis griseus)[10029]	G3HH02	3	6	186.36	0.05292479
Uniprot:(Cricetulus barabensis griseus)[10029]	G3HH30	8	19	649.42	0.23417722
Uniprot:(Cricetulus barabensis griseus)[10029]	G3HH47	2	6	235.58	0.23076923
Uniprot:(Cricetulus barabensis griseus)[10029]	G3HH92	1	2	123.93	0.08988764
Uniprot:(Cricetulus barabensis griseus)[10029]	G3HHC7	1	1	20.4	0.03269755
Uniprot:(Cricetulus barabensis griseus)[10029]	G3HHR3	14	31	1182.3	0.3304721
Uniprot:(Cricetulus barabensis griseus)[10029]	G3HHW0	1	1	20.67	0.00970874
Uniprot:(Cricetulus barabensis griseus)[10029]	G3HI29	1	2	51.14	0.03296703
Uniprot:(Cricetulus barabensis griseus)[10029]	G3HIB5	1	1	21.69	0.0212766
Uniprot:(Cricetulus barabensis griseus)[10029]	G3HIG4	1	2	44.33	0.02480916
Uniprot:(Cricetulus barabensis griseus)[10029]	G3HIK2	1	1	22.74	0.01407035
Uniprot:(Cricetulus barabensis griseus)[10029]	G3HIK9	1	1	25.6	0.14159292
Uniprot:(Cricetulus barabensis griseus)[10029]	G3HIM1	9	24	842.3	0.03471338
Uniprot:(Cricetulus barabensis griseus)[10029]	G3HIM4	1	2	93.62	0.08900524
Uniprot:(Cricetulus barabensis griseus)[10029]	G3HIQ0	1	1	24.13	0.01108033
Uniprot:(Cricetulus barabensis griseus)[10029]	G3HIQ1	1	7	259.54	0.1097561
Uniprot:(Cricetulus barabensis griseus)[10029]	G3HIT7	1	1	32.21	0.01307465
Uniprot:(Cricetulus barabensis griseus)[10029]	G3HIU1	1	1	21.8	0.01239669
Uniprot:(Cricetulus barabensis griseus)[10029]	G3HIV4	1	1	21.46	0.04972376
Uniprot:(Cricetulus barabensis griseus)[10029]	G3HIX6	1	4	232.42	0.03368421
Uniprot:(Cricetulus barabensis griseus)[10029]	G3HJ12	1	1	21.96	0.09836066
Uniprot:(Cricetulus barabensis griseus)[10029]	G3HJ55	1	1	20.88	0.01818182
Uniprot:(Cricetulus barabensis griseus)[10029]	G3HJ73	1	1	21.09	0.01487603
Uniprot:(Cricetulus barabensis griseus)[10029]	G3HJ89	2	3	77.82	0.00765306
Uniprot:(Cricetulus barabensis griseus)[10029]	G3HJ99	2	2	45.63	0.01477833
Uniprot:(Cricetulus barabensis griseus)[10029]	G3HJB0	2	4	107.46	0.04892966
Uniprot:(Cricetulus barabensis griseus)[10029]	G3HJB1	1	2	90.38	0.04700855

Uniprot:(Cricetulus barabensis griseus)[10029]	G3HJG6	1	2	53.98	0.03038674
Uniprot:(Cricetulus barabensis griseus)[10029]	G3HJH6	1	1	20.28	0.08695652
Uniprot:(Cricetulus barabensis griseus)[10029]	G3HJJ1	1	2	51.19	0.04469274
Uniprot:(Cricetulus barabensis griseus)[10029]	G3HJJ2	1	1	21.48	0.01660281
Uniprot:(Cricetulus barabensis griseus)[10029]	G3HJM2	1	1	23.69	0.01735016
Uniprot:(Cricetulus barabensis griseus)[10029]	G3HJS0	2	2	42.98	0.01402525
Uniprot:(Cricetulus barabensis griseus)[10029]	G3HJV4	1	3	71.65	0.05263158
Uniprot:(Cricetulus barabensis griseus)[10029]	G3HK65	1	1	20.33	0.0037594
Uniprot:(Cricetulus barabensis griseus)[10029]	G3HK90	4	8	285.81	0.1779661
Uniprot:(Cricetulus barabensis griseus)[10029]	G3HKG9	6	15	452	0.20714286
Uniprot:(Cricetulus barabensis griseus)[10029]	G3HKQ5	1	3	67.82	0.02605863
Uniprot:(Cricetulus barabensis griseus)[10029]	G3HKQ7	4	5	147.33	0.03
Uniprot:(Cricetulus barabensis griseus)[10029]	G3HKY4	1	1	21.39	0.02446483
Uniprot:(Cricetulus barabensis griseus)[10029]	G3HKZ1	6	14	575.34	0.32653061
Uniprot:(Cricetulus barabensis griseus)[10029]	G3HL04	1	2	47.34	0.09285714
Uniprot:(Cricetulus barabensis griseus)[10029]	G3HL05	1	1	22.65	0.01196172
Uniprot:(Cricetulus barabensis griseus)[10029]	G3HL07	1	1	20.85	0.007034
Uniprot:(Cricetulus barabensis griseus)[10029]	G3HL22	1	1	23.26	0.01123596
Uniprot:(Cricetulus barabensis griseus)[10029]	G3HLK3	2	6	167.1	0.02533784
Uniprot:(Cricetulus barabensis griseus)[10029]	G3HLL4	1	1	29.92	0.01028807
Uniprot:(Cricetulus barabensis griseus)[10029]	G3HLN7	1	1	25.45	0.03021148
Uniprot:(Cricetulus barabensis griseus)[10029]	G3HLR7	1	1	28.05	0.03581267
Uniprot:(Cricetulus barabensis griseus)[10029]	G3HLS2	3	4	138.39	0.10655738
Uniprot:(Cricetulus barabensis griseus)[10029]	G3HLT3	3	3	123.51	0.20792079
Uniprot:(Cricetulus barabensis griseus)[10029]	G3HLV1	1	2	50.68	0.01376936
Uniprot:(Cricetulus barabensis griseus)[10029]	G3HLV6	4	9	232.13	0.05164993
Uniprot:(Cricetulus barabensis griseus)[10029]	G3HM03	3	6	173.55	0.12355212
Uniprot:(Cricetulus barabensis griseus)[10029]	G3HM88	1	1	21.05	0.01584507
Uniprot:(Cricetulus barabensis griseus)[10029]	G3HM99	2	14	650.43	0.08300395
Uniprot:(Cricetulus barabensis griseus)[10029]	G3HMA1	3	5	177.06	0.02930728
Uniprot:(Cricetulus barabensis griseus)[10029]	G3HMB8	2	4	96.95	0.18965517
Uniprot:(Cricetulus barabensis griseus)[10029]	G3HMG4	5	11	417.48	0.12009238
Uniprot:(Cricetulus barabensis griseus)[10029]	G3HMM2	1	1	25.25	0.04090909
Uniprot:(Cricetulus barabensis griseus)[10029]	G3HMQ0	3	4	119.92	0.05789474
Uniprot:(Cricetulus barabensis griseus)[10029]	G3HMV7	8	25	808.43	0.1991342
Uniprot:(Cricetulus barabensis griseus)[10029]	G3HMW9	1	1	20.24	0.02766798
Uniprot:(Cricetulus barabensis griseus)[10029]	G3HN02	1	2	62.66	0.00865333
Uniprot:(Cricetulus barabensis griseus)[10029]	G3HN14	11	31	1077.2	0.05895901
Uniprot:(Cricetulus barabensis griseus)[10029]	G3HN77	1	2	80.74	0.02099237
Uniprot:(Cricetulus barabensis griseus)[10029]	G3HN88	3	4	135.6	0.08649789
Uniprot:(Cricetulus barabensis griseus)[10029]	G3HNC5	1	2	50.63	0.00518519
Uniprot:(Cricetulus barabensis griseus)[10029]	G3HND4	1	2	63.99	0.07894737
Uniprot:(Cricetulus barabensis griseus)[10029]	G3HNG5	1	1	32.74	0.02380952
Uniprot:(Cricetulus barabensis griseus)[10029]	G3HNJ2	1	1	20.88	0.01724138
Uniprot:(Cricetulus barabensis griseus)[10029]	G3HNJ3	8	21	738.96	0.20357942

Uniprot:(Cricetulus barabensis griseus)[10029]	G3HNJ4	1	1	23.18	0.01260504
Uniprot:(Cricetulus barabensis griseus)[10029]	G3HNK4	1	2	65.12	0.02097902
Uniprot:(Cricetulus barabensis griseus)[10029]	G3HNN9	1	1	22.33	0.03
Uniprot:(Cricetulus barabensis griseus)[10029]	G3HNT9	3	9	314.14	0.30714286
Uniprot:(Cricetulus barabensis griseus)[10029]	G3HNW4	1	1	23.8	0.00970874
Uniprot:(Cricetulus barabensis griseus)[10029]	G3HNY4	1	3	75.72	0.05298013
Uniprot:(Cricetulus barabensis griseus)[10029]	G3HNY5	3	12	382.2	0.15289256
Uniprot:(Cricetulus barabensis griseus)[10029]	G3HP75	1	3	94.42	0.0206379
Uniprot:(Cricetulus barabensis griseus)[10029]	G3HP82	1	1	20.1	0.0056338
Uniprot:(Cricetulus barabensis griseus)[10029]	G3HPE9	1	1	25.64	0.02240896
Uniprot:(Cricetulus barabensis griseus)[10029]	G3HPH9	1	1	54.7	0.0625
Uniprot:(Cricetulus barabensis griseus)[10029]	G3HPM5	1	1	34.51	0.05300353
Uniprot:(Cricetulus barabensis griseus)[10029]	G3HPZ5	2	6	225.51	0.08852459
Uniprot:(Cricetulus barabensis griseus)[10029]	G3HQ29	1	1	22.43	0.01664533
Uniprot:(Cricetulus barabensis griseus)[10029]	G3HQ69	2	3	79.18	0.03272727
Uniprot:(Cricetulus barabensis griseus)[10029]	G3HQG4	1	1	21.99	0.00582242
Uniprot:(Cricetulus barabensis griseus)[10029]	G3HQH8	1	3	95.6	0.07462687
Uniprot:(Cricetulus barabensis griseus)[10029]	G3HQL6	7	21	629.42	0.11504425
Uniprot:(Cricetulus barabensis griseus)[10029]	G3HQM6	26	76	2566	0.33499377
Uniprot:(Cricetulus barabensis griseus)[10029]	G3HQP6	1	3	79.27	0.0625
Uniprot:(Cricetulus barabensis griseus)[10029]	G3HQP8	3	10	309.13	0.09234234
Uniprot:(Cricetulus barabensis griseus)[10029]	G3HQV2	1	1	29.37	0.02347418
Uniprot:(Cricetulus barabensis griseus)[10029]	G3HQW8	1	1	27.15	0.03116147
Uniprot:(Cricetulus barabensis griseus)[10029]	G3HQY2	7	23	794.08	0.14446953
Uniprot:(Cricetulus barabensis griseus)[10029]	G3HQY9	1	1	22.45	0.03293413
Uniprot:(Cricetulus barabensis griseus)[10029]	G3HR08	3	9	270.36	0.06926407
Uniprot:(Cricetulus barabensis griseus)[10029]	G3HR96	2	2	64.93	0.05054945
Uniprot:(Cricetulus barabensis griseus)[10029]	G3HRA1	1	6	140.03	0.01066098
Uniprot:(Cricetulus barabensis griseus)[10029]	G3HRB8	1	1	21.39	0.01213592
Uniprot:(Cricetulus barabensis griseus)[10029]	G3HRD3	2	3	64.29	0.03466205
Uniprot:(Cricetulus barabensis griseus)[10029]	G3HRF0	1	1	21.69	0.03278689
Uniprot:(Cricetulus barabensis griseus)[10029]	G3HRI4	1	1	22.59	0.03294893
Uniprot:(Cricetulus barabensis griseus)[10029]	G3HRJ2	1	1	27.51	0.00708801
Uniprot:(Cricetulus barabensis griseus)[10029]	G3HRK0	5	8	280.01	0.13451777
Uniprot:(Cricetulus barabensis griseus)[10029]	G3HRK9	5	13	453.35	0.14285714
Uniprot:(Cricetulus barabensis griseus)[10029]	G3HRN1	1	1	20.56	0.00907151
Uniprot:(Cricetulus barabensis griseus)[10029]	G3HRP2	1	3	95.21	0.05494506
Uniprot:(Cricetulus barabensis griseus)[10029]	G3HRP3	1	2	54	0.03514377
Uniprot:(Cricetulus barabensis griseus)[10029]	G3HRT4	1	1	20	0.0033358
Uniprot:(Cricetulus barabensis griseus)[10029]	G3HS15	1	3	73.28	0.02811245
Uniprot:(Cricetulus barabensis griseus)[10029]	G3HS71	1	2	50.76	0.01337296
Uniprot:(Cricetulus barabensis griseus)[10029]	G3HS88	1	5	156.17	0.0591716
Uniprot:(Cricetulus barabensis griseus)[10029]	G3HSA7	1	3	60.6	0.05681818
Uniprot:(Cricetulus barabensis griseus)[10029]	G3HSB7	1	1	21.41	0.05027933
Uniprot:(Cricetulus barabensis griseus)[10029]	G3HSE4	3	7	153.83	0.09885932

Uniprot:(Cricetulus barabensis griseus)[10029]	G3HSF3	4	8	215.22	0.23076923
Uniprot:(Cricetulus barabensis griseus)[10029]	G3HSK6	1	1	20.74	0.01659751
Uniprot:(Cricetulus barabensis griseus)[10029]	G3HSL4	14	46	1391.7	0.20068807
Uniprot:(Cricetulus barabensis griseus)[10029]	G3HSM3	3	4	96.96	0.0363901
Uniprot:(Cricetulus barabensis griseus)[10029]	G3HSM8	2	3	69.13	0.02325581
Uniprot:(Cricetulus barabensis griseus)[10029]	G3HSV9	1	1	22.41	0.02949062
Uniprot:(Cricetulus barabensis griseus)[10029]	G3HSW2	1	6	143.77	0.05240175
Uniprot:(Cricetulus barabensis griseus)[10029]	G3HSX8	7	36	1234.9	0.22493225
Uniprot:(Cricetulus barabensis griseus)[10029]	G3HSZ6	2	3	80.5	0.07017544
Uniprot:(Cricetulus barabensis griseus)[10029]	G3HT06	1	14	545.9	0.10714286
Uniprot:(Cricetulus barabensis griseus)[10029]	G3HT14	1	1	22.76	0.02118644
Uniprot:(Cricetulus barabensis griseus)[10029]	G3HT74	1	1	20.94	0.25925926
Uniprot:(Cricetulus barabensis griseus)[10029]	G3HTE5	5	6	177.93	0.06427819
Uniprot:(Cricetulus barabensis griseus)[10029]	G3HTF5	1	1	37	0.07142857
Uniprot:(Cricetulus barabensis griseus)[10029]	G3HTF8	1	1	28.83	0.03669725
Uniprot:(Cricetulus barabensis griseus)[10029]	G3HTG9	5	5	150.38	0.15436242
Uniprot:(Cricetulus barabensis griseus)[10029]	G3HTJ2	1	1	24.37	0.03672316
Uniprot:(Cricetulus barabensis griseus)[10029]	G3HTL9	1	1	23.23	0.00895255
Uniprot:(Cricetulus barabensis griseus)[10029]	G3HTM1	1	1	24.91	0.01067073
Uniprot:(Cricetulus barabensis griseus)[10029]	G3HTR9	1	2	53.44	0.01541096
Uniprot:(Cricetulus barabensis griseus)[10029]	G3HTX8	1	1	20.52	0.12612613
Uniprot:(Cricetulus barabensis griseus)[10029]	G3HTZ1	1	1	24	0.01372998
Uniprot:(Cricetulus barabensis griseus)[10029]	G3HU10	3	11	321.66	0.12605042
Uniprot:(Cricetulus barabensis griseus)[10029]	G3HU28	4	8	224.69	0.13178295
Uniprot:(Cricetulus barabensis griseus)[10029]	G3HU34	1	7	154.78	0.07086614
Uniprot:(Cricetulus barabensis griseus)[10029]	G3HU51	7	22	645.47	0.20289855
Uniprot:(Cricetulus barabensis griseus)[10029]	G3HU79	1	1	23.01	0.01564945
Uniprot:(Cricetulus barabensis griseus)[10029]	G3HUD0	1	1	26.8	0.14754098
Uniprot:(Cricetulus barabensis griseus)[10029]	G3HUD4	1	1	21.39	0.11764706
Uniprot:(Cricetulus barabensis griseus)[10029]	G3HUM5	1	4	115.85	0.02063107
Uniprot:(Cricetulus barabensis griseus)[10029]	G3HUT8	1	1	22.8	0.11564626
Uniprot:(Cricetulus barabensis griseus)[10029]	G3HUU6	1	2	41.85	0.07
Uniprot:(Cricetulus barabensis griseus)[10029]	G3HUU7	1	1	20.49	0.17525773
Uniprot:(Cricetulus barabensis griseus)[10029]	G3HUW5	1	1	24.76	0.01736111
Uniprot:(Cricetulus barabensis griseus)[10029]	G3HV18	1	2	89.3	0.1119403
Uniprot:(Cricetulus barabensis griseus)[10029]	G3HV46	1	1	20.51	0.0486618
Uniprot:(Cricetulus barabensis griseus)[10029]	G3HV83	3	5	121.38	0.13356164
Uniprot:(Cricetulus barabensis griseus)[10029]	G3HVC8	1	1	24.9	0.01455027
Uniprot:(Cricetulus barabensis griseus)[10029]	G3HVP2	1	4	158.75	0.04591837
Uniprot:(Cricetulus barabensis griseus)[10029]	G3HVP8	1	1	24.19	0.01182796
Uniprot:(Cricetulus barabensis griseus)[10029]	G3HW06	3	4	113.43	0.0785124
Uniprot:(Cricetulus barabensis griseus)[10029]	G3HW48	1	1	22.54	0.02238806
Uniprot:(Cricetulus barabensis griseus)[10029]	G3HW69	1	1	20.4	0.0410628
Uniprot:(Cricetulus barabensis griseus)[10029]	G3HWB9	1	1	25.51	0.00792752
Uniprot:(Cricetulus barabensis griseus)[10029]	G3HWC3	9	19	676.64	0.16552901

Uniprot:(Cricetulus barabensis griseus)[10029]	G3HWE4	3	9	449.84	0.06500692
Uniprot:(Cricetulus barabensis griseus)[10029]	G3HWE7	4	8	483.44	0.18390805
Uniprot:(Cricetulus barabensis griseus)[10029]	G3HWJ1	1	2	44.46	0.00709939
Uniprot:(Cricetulus barabensis griseus)[10029]	G3HWJ2	1	4	201.15	0.02638889
Uniprot:(Cricetulus barabensis griseus)[10029]	G3HWJ3	18	35	1181.1	0.11196911
Uniprot:(Cricetulus barabensis griseus)[10029]	G3HX36	1	1	20.76	0.00729927
Uniprot:(Cricetulus barabensis griseus)[10029]	G3HX39	5	9	338.64	0.25641026
Uniprot:(Cricetulus barabensis griseus)[10029]	G3HX67	2	2	52.23	0.03611738
Uniprot:(Cricetulus barabensis griseus)[10029]	G3HX78	1	2	45.22	0.01044932
Uniprot:(Cricetulus barabensis griseus)[10029]	G3HXF7	3	15	504.63	0.1147541
Uniprot:(Cricetulus barabensis griseus)[10029]	G3HXP3	1	1	20.38	0.06896552
Uniprot:(Cricetulus barabensis griseus)[10029]	G3HXT3	1	1	25.94	0.03461539
Uniprot:(Cricetulus barabensis griseus)[10029]	G3HXW9	1	1	21.44	0.01595745
Uniprot:(Cricetulus barabensis griseus)[10029]	G3HXY1	1	1	20.84	0.03343465
Uniprot:(Cricetulus barabensis griseus)[10029]	G3HXZ0	1	1	22.43	0.02597403
Uniprot:(Cricetulus barabensis griseus)[10029]	G3HY08	1	1	25.88	0.09565217
Uniprot:(Cricetulus barabensis griseus)[10029]	G3HY14	1	1	23.81	0.0141129
Uniprot:(Cricetulus barabensis griseus)[10029]	G3HYB7	3	8	210.03	0.06212425
Uniprot:(Cricetulus barabensis griseus)[10029]	G3HYF4	1	1	20.81	0.07070707
Uniprot:(Cricetulus barabensis griseus)[10029]	G3HYG2	1	1	23.5	0.01719902
Uniprot:(Cricetulus barabensis griseus)[10029]	G3HYJ8	1	1	24.67	0.09803922
Uniprot:(Cricetulus barabensis griseus)[10029]	G3HYJ9	7	11	387.33	0.21843003
Uniprot:(Cricetulus barabensis griseus)[10029]	G3HYP6	2	3	77.01	0.05673759
Uniprot:(Cricetulus barabensis griseus)[10029]	G3HYS7	1	1	21.81	0.02288732
Uniprot:(Cricetulus barabensis griseus)[10029]	G3HYT5	2	2	71.71	0.11656442
Uniprot:(Cricetulus barabensis griseus)[10029]	G3HYZ5	2	7	204.76	0.0565371
Uniprot:(Cricetulus barabensis griseus)[10029]	G3HZ42	3	8	256.88	0.0728972
Uniprot:(Cricetulus barabensis griseus)[10029]	G3HZ75	1	1	21.77	0.03076923
Uniprot:(Cricetulus barabensis griseus)[10029]	G3HZD1	1	1	27.48	0.00395257
Uniprot:(Cricetulus barabensis griseus)[10029]	G3HZE3	1	6	233.46	0.02808989
Uniprot:(Cricetulus barabensis griseus)[10029]	G3HZG3	1	1	22.47	0.0368272
Uniprot:(Cricetulus barabensis griseus)[10029]	G3HZV0	2	3	70.14	0.15789474
Uniprot:(Cricetulus barabensis griseus)[10029]	G3HZX6	2	4	153.54	0.16393443
Uniprot:(Cricetulus barabensis griseus)[10029]	G3I015	3	4	118.02	0.10583942
Uniprot:(Cricetulus barabensis griseus)[10029]	G3I027	1	2	124.37	0.01983003
Uniprot:(Cricetulus barabensis griseus)[10029]	G3I064	3	7	243.72	0.0286017
Uniprot:(Cricetulus barabensis griseus)[10029]	G3I068	1	1	20.55	0.0989011
Uniprot:(Cricetulus barabensis griseus)[10029]	G3I073	1	4	119.76	0.03846154
Uniprot:(Cricetulus barabensis griseus)[10029]	G3I075	1	2	68.95	0.07216495
Uniprot:(Cricetulus barabensis griseus)[10029]	G3I0F7	2	4	137	0.08192771
Uniprot:(Cricetulus barabensis griseus)[10029]	G3I0I7	1	2	94.25	0.05555556
Uniprot:(Cricetulus barabensis griseus)[10029]	G3I0M1	2	9	189.08	0.00985222
Uniprot:(Cricetulus barabensis griseus)[10029]	G3I0M7	1	1	21.54	0.02011494
Uniprot:(Cricetulus barabensis griseus)[10029]	G3I0R3	2	2	64.19	0.04987531
Uniprot:(Cricetulus barabensis griseus)[10029]	G3I0W1	1	6	153.14	0.07194245

Uniprot:(Cricetulus barabensis griseus)[10029]	G3I129	3	7	238.48	0.05581395
Uniprot:(Cricetulus barabensis griseus)[10029]	G3I167	1	1	20.65	0.00783546
Uniprot:(Cricetulus barabensis griseus)[10029]	G3I183	1	2	43.09	0.01358412
Uniprot:(Cricetulus barabensis griseus)[10029]	G3I1B6	1	2	46.61	0.02369668
Uniprot:(Cricetulus barabensis griseus)[10029]	G3I1H5	7	37	1461.3	0.22374429
Uniprot:(Cricetulus barabensis griseus)[10029]	G3I1J3	1	14	306.75	0.01208054
Uniprot:(Cricetulus barabensis griseus)[10029]	G3I1P0	1	2	75.25	0.06214689
Uniprot:(Cricetulus barabensis griseus)[10029]	G3I1P5	4	12	494.55	0.46491228
Uniprot:(Cricetulus barabensis griseus)[10029]	G3I1R2	2	4	112.51	0.08127209
Uniprot:(Cricetulus barabensis griseus)[10029]	G3I1V3	6	12	413.67	0.02775543
Uniprot:(Cricetulus barabensis griseus)[10029]	G3I1V4	1	2	74.95	0.02533784
Uniprot:(Cricetulus barabensis griseus)[10029]	G3I1Y9	6	15	426.98	0.24096386
Uniprot:(Cricetulus barabensis griseus)[10029]	G3I207	1	1	23.92	0.01298701
Uniprot:(Cricetulus barabensis griseus)[10029]	G3I216	2	4	143.77	0.10160428
Uniprot:(Cricetulus barabensis griseus)[10029]	G3I230	1	3	108.66	0.04219409
Uniprot:(Cricetulus barabensis griseus)[10029]	G3I255	8	35	1403.3	0.28534031
Uniprot:(Cricetulus barabensis griseus)[10029]	G3I256	1	3	64.3	0.02189781
Uniprot:(Cricetulus barabensis griseus)[10029]	G3I278	9	22	874.54	0.06802721
Uniprot:(Cricetulus barabensis griseus)[10029]	G3I295	1	7	161.7	0.00632911
Uniprot:(Cricetulus barabensis griseus)[10029]	G3I2A7	1	1	20.55	0.01104295
Uniprot:(Cricetulus barabensis griseus)[10029]	G3I2C1	1	1	21.3	0.04761905
Uniprot:(Cricetulus barabensis griseus)[10029]	G3I2C8	1	2	46.28	0.01358234
Uniprot:(Cricetulus barabensis griseus)[10029]	G3I2H0	3	6	220.14	0.05420054
Uniprot:(Cricetulus barabensis griseus)[10029]	G3I2K6	3	13	454.85	0.15544042
Uniprot:(Cricetulus barabensis griseus)[10029]	G3I2M0	1	2	62.94	0.04845815
Uniprot:(Cricetulus barabensis griseus)[10029]	G3I2M1	4	5	121.76	0.07532957
Uniprot:(Cricetulus barabensis griseus)[10029]	G3I2M2	1	1	23.46	0.032
Uniprot:(Cricetulus barabensis griseus)[10029]	G3I2R2	1	1	23.01	0.00970874
Uniprot:(Cricetulus barabensis griseus)[10029]	G3I2T9	2	5	143.15	0.02503129
Uniprot:(Cricetulus barabensis griseus)[10029]	G3I2V7	1	1	21.25	0.03470032
Uniprot:(Cricetulus barabensis griseus)[10029]	G3I323	1	1	22.89	0.024
Uniprot:(Cricetulus barabensis griseus)[10029]	G3I342	1	1	24.72	0.00958904
Uniprot:(Cricetulus barabensis griseus)[10029]	G3I366	1	1	27.81	0.02702703
Uniprot:(Cricetulus barabensis griseus)[10029]	G3I3D5	1	1	24.22	0.16129032
Uniprot:(Cricetulus barabensis griseus)[10029]	G3I3D7	1	2	46.92	0.01311475
Uniprot:(Cricetulus barabensis griseus)[10029]	G3I3G8	4	11	418.23	0.11275964
Uniprot:(Cricetulus barabensis griseus)[10029]	G3I3H2	5	14	649.34	0.80869565
Uniprot:(Cricetulus barabensis griseus)[10029]	G3I3H9	1	1	46.94	0.02290076
Uniprot:(Cricetulus barabensis griseus)[10029]	G3I3I5	1	1	20.16	0.06666667
Uniprot:(Cricetulus barabensis griseus)[10029]	G3I3J5	1	1	37.29	0.09375
Uniprot:(Cricetulus barabensis griseus)[10029]	G3I3K5	1	1	24	0.01453488
Uniprot:(Cricetulus barabensis griseus)[10029]	G3I3N5	1	1	35.37	0.02094241
Uniprot:(Cricetulus barabensis griseus)[10029]	G3I3U5	3	10	365.27	0.12589928
Uniprot:(Cricetulus barabensis griseus)[10029]	G3I3X4	7	33	1156.8	0.16483517
Uniprot:(Cricetulus barabensis griseus)[10029]	G3I3X6	1	2	130.7	0.1097561

Uniprot:(Cricetulus barabensis griseus)[10029]	G3I3Y6	6	42	1794.4	0.36651584
Uniprot:(Cricetulus barabensis griseus)[10029]	G3I3Z0	1	1	23.2	0.00562701
Uniprot:(Cricetulus barabensis griseus)[10029]	G3I412	1	1	24.78	0.02535211
Uniprot:(Cricetulus barabensis griseus)[10029]	G3I436	1	2	54.5	0.0448718
Uniprot:(Cricetulus barabensis griseus)[10029]	G3I485	1	1	22.32	0.02631579
Uniprot:(Cricetulus barabensis griseus)[10029]	G3I4D4	3	18	511.05	0.12355212
Uniprot:(Cricetulus barabensis griseus)[10029]	G3I4E6	1	1	27	0.03813559
Uniprot:(Cricetulus barabensis griseus)[10029]	G3I4E8	4	6	247.07	0.31060606
Uniprot:(Cricetulus barabensis griseus)[10029]	G3I4G1	2	3	92.82	0.01726727
Uniprot:(Cricetulus barabensis griseus)[10029]	G3I4H6	8	27	1406.3	0.3543956
Uniprot:(Cricetulus barabensis griseus)[10029]	G3I4I3	1	1	22.08	0.0136876
Uniprot:(Cricetulus barabensis griseus)[10029]	G3I4J9	1	2	52.06	0.04697987
Uniprot:(Cricetulus barabensis griseus)[10029]	G3I4K1	2	8	189.65	0.02
Uniprot:(Cricetulus barabensis griseus)[10029]	G3I4K2	2	2	51.89	0.05194805
Uniprot:(Cricetulus barabensis griseus)[10029]	G3I4U1	1	1	22.88	0.04658385
Uniprot:(Cricetulus barabensis griseus)[10029]	G3I4V4	1	1	26.52	0.01210428
Uniprot:(Cricetulus barabensis griseus)[10029]	G3I4W7	7	12	538.32	0.18872549
Uniprot:(Cricetulus barabensis griseus)[10029]	G3I4Z7	2	23	903.28	0.17777778
Uniprot:(Cricetulus barabensis griseus)[10029]	G3I539	1	1	27.56	0.08064516
Uniprot:(Cricetulus barabensis griseus)[10029]	G3I581	1	2	41.29	0.09859155
Uniprot:(Cricetulus barabensis griseus)[10029]	G3I595	1	2	55.6	0.03021978
Uniprot:(Cricetulus barabensis griseus)[10029]	G3I596	2	2	61.76	0.24489796
Uniprot:(Cricetulus barabensis griseus)[10029]	G3I597	1	2	50.62	0.02465753
Uniprot:(Cricetulus barabensis griseus)[10029]	G3I598	1	1	20.12	0.04020101
Uniprot:(Cricetulus barabensis griseus)[10029]	G3I5A8	1	2	40.56	0.06060606
Uniprot:(Cricetulus barabensis griseus)[10029]	G3I5G8	1	1	24.24	0.1171875
Uniprot:(Cricetulus barabensis griseus)[10029]	G3I5H1	2	6	161.36	0.07657658
Uniprot:(Cricetulus barabensis griseus)[10029]	G3I5H3	1	1	30.17	0.03915663
Uniprot:(Cricetulus barabensis griseus)[10029]	G3I5K2	1	1	21.22	0.04669261
Uniprot:(Cricetulus barabensis griseus)[10029]	G3I5L3	3	7	228.42	0.07803468
Uniprot:(Cricetulus barabensis griseus)[10029]	G3I5L5	2	6	145.63	0.03516484
Uniprot:(Cricetulus barabensis griseus)[10029]	G3I5N6	1	1	21.61	0.03149606
Uniprot:(Cricetulus barabensis griseus)[10029]	G3I5Q9	2	6	225.1	0.09633028
Uniprot:(Cricetulus barabensis griseus)[10029]	G3I5R2	1	2	64.7	0.13043478
Uniprot:(Cricetulus barabensis griseus)[10029]	G3I5T9	4	7	188.58	0.34210526
Uniprot:(Cricetulus barabensis griseus)[10029]	G3I5X5	1	1	29.68	0.03684211
Uniprot:(Cricetulus barabensis griseus)[10029]	G3I5Z5	3	6	184.6	0.07594937
Uniprot:(Cricetulus barabensis griseus)[10029]	G3I621	1	4	130.89	0.01910828
Uniprot:(Cricetulus barabensis griseus)[10029]	G3I623	1	6	131.08	0.00679502
Uniprot:(Cricetulus barabensis griseus)[10029]	G3I664	2	4	228.46	0.0589391
Uniprot:(Cricetulus barabensis griseus)[10029]	G3I683	2	4	97.01	0.06603774
Uniprot:(Cricetulus barabensis griseus)[10029]	G3I692	1	5	212.39	0.0231579
Uniprot:(Cricetulus barabensis griseus)[10029]	G3I6A8	1	1	20.01	0.03293413
Uniprot:(Cricetulus barabensis griseus)[10029]	G3I6P0	1	1	21.71	0.16666667
Uniprot:(Cricetulus barabensis griseus)[10029]	G3I6P1	2	14	476.36	0.10144928

Uniprot:(Cricetulus barabensis griseus)[10029]	G3I6T1	4	17	614.24	0.09059829
Uniprot:(Cricetulus barabensis griseus)[10029]	G3I6W5	1	2	105.04	0.09448819
Uniprot:(Cricetulus barabensis griseus)[10029]	G3I6Z8	1	2	40.82	0.0617284
Uniprot:(Cricetulus barabensis griseus)[10029]	G3I715	1	1	20.5	0.02298851
Uniprot:(Cricetulus barabensis griseus)[10029]	G3I735	1	2	46	0.09333333
Uniprot:(Cricetulus barabensis griseus)[10029]	G3I737	1	4	133.52	0.08264463
Uniprot:(Cricetulus barabensis griseus)[10029]	G3I740	1	2	93.74	0.02734375
Uniprot:(Cricetulus barabensis griseus)[10029]	G3I780	1	1	21.93	0.01476015
Uniprot:(Cricetulus barabensis griseus)[10029]	G3I7A8	2	3	76.94	0.0184874
Uniprot:(Cricetulus barabensis griseus)[10029]	G3I7D8	1	1	22.56	0.0625
Uniprot:(Cricetulus barabensis griseus)[10029]	G3I7Q1	1	1	27.23	0.02272727
Uniprot:(Cricetulus barabensis griseus)[10029]	G3I7U4	2	3	68.65	0.02796272
Uniprot:(Cricetulus barabensis griseus)[10029]	G3I842	1	1	23.14	0.06097561
Uniprot:(Cricetulus barabensis griseus)[10029]	G3I877	8	19	517.37	0.13812155
Uniprot:(Cricetulus barabensis griseus)[10029]	G3I8C0	1	1	22.17	0.01392758
Uniprot:(Cricetulus barabensis griseus)[10029]	G3I8F7	1	1	21.64	0.04218362
Uniprot:(Cricetulus barabensis griseus)[10029]	G3I8J5	1	1	20.41	0.01886793
Uniprot:(Cricetulus barabensis griseus)[10029]	G3I8L4	1	2	60.83	0.02894737
Uniprot:(Cricetulus barabensis griseus)[10029]	G3I8P3	1	1	21.85	0.00928793
Uniprot:(Cricetulus barabensis griseus)[10029]	G3I8P7	3	6	175.68	0.07383966
Uniprot:(Cricetulus barabensis griseus)[10029]	G3I8R9	26	127	5134.5	0.5030581
Uniprot:(Cricetulus barabensis griseus)[10029]	G3I8U9	1	2	43.53	0.04958678
Uniprot:(Cricetulus barabensis griseus)[10029]	G3I8V4	1	1	32.93	0.02267574
Uniprot:(Cricetulus barabensis griseus)[10029]	G3I8Y4	1	1	22.12	0.06422018
Uniprot:(Cricetulus barabensis griseus)[10029]	G3I936	1	3	76.16	0.04697987
Uniprot:(Cricetulus barabensis griseus)[10029]	G3I948	2	7	318.45	0.13071895
Uniprot:(Cricetulus barabensis griseus)[10029]	G3I952	5	11	483.93	0.43884892
Uniprot:(Cricetulus barabensis griseus)[10029]	G3I968	1	1	25.87	0.05594406
Uniprot:(Cricetulus barabensis griseus)[10029]	G3I973	4	9	299.18	0.04204204
Uniprot:(Cricetulus barabensis griseus)[10029]	G3I9A3	2	2	72.93	0.28571429
Uniprot:(Cricetulus barabensis griseus)[10029]	G3I9B2	1	2	47.44	0.006917
Uniprot:(Cricetulus barabensis griseus)[10029]	G3I9D3	2	3	101.31	0.06859206
Uniprot:(Cricetulus barabensis griseus)[10029]	G3I9F0	3	3	84.89	0.03448276
Uniprot:(Cricetulus barabensis griseus)[10029]	G3I9G7	4	11	374.49	0.1598513
Uniprot:(Cricetulus barabensis griseus)[10029]	G3I9P1	2	3	65.62	0.09734513
Uniprot:(Cricetulus barabensis griseus)[10029]	G3I9R5	1	1	21.47	0.00996933
Uniprot:(Cricetulus barabensis griseus)[10029]	G3I9V1	1	1	20.92	0.04314721
Uniprot:(Cricetulus barabensis griseus)[10029]	G3IA01	1	1	21.05	0.01820728
Uniprot:(Cricetulus barabensis griseus)[10029]	G3IA08	1	7	171.03	0.02325581
Uniprot:(Cricetulus barabensis griseus)[10029]	G3IA91	1	4	142.9	0.0608365
Uniprot:(Cricetulus barabensis griseus)[10029]	G3IA94	1	2	56.55	0.06395349
Uniprot:(Cricetulus barabensis griseus)[10029]	G3IAD2	1	4	122.8	0.08588957
Uniprot:(Cricetulus barabensis griseus)[10029]	G3IAE3	1	2	63.44	0.14545455
Uniprot:(Cricetulus barabensis griseus)[10029]	G3IAG0	1	2	44.41	0.02568218
Uniprot:(Cricetulus barabensis griseus)[10029]	G3IAI6	6	14	473.78	0.3

Uniprot:(Cricetulus barabensis griseus)[10029]	G3IAQ0	14	85	3324.4	0.47086247
Uniprot:(Cricetulus barabensis griseus)[10029]	G3IAS8	2	2	63.83	0.01031553
Uniprot:(Cricetulus barabensis griseus)[10029]	G3IAV1	1	1	28.54	0.03285421
Uniprot:(Cricetulus barabensis griseus)[10029]	G3IAW1	1	2	50.9	0.01917808
Uniprot:(Cricetulus barabensis griseus)[10029]	G3IAX3	8	55	2805.4	0.19305857
Uniprot:(Cricetulus barabensis griseus)[10029]	G3IB32	1	1	23.57	0.01694915
Uniprot:(Cricetulus barabensis griseus)[10029]	G3IB34	1	4	85.08	0.00481283
Uniprot:(Cricetulus barabensis griseus)[10029]	G3IB46	1	1	24.64	0.01353384
Uniprot:(Cricetulus barabensis griseus)[10029]	G3IB99	1	2	47.33	0.04444444
Uniprot:(Cricetulus barabensis griseus)[10029]	G3IBF1	1	1	28.49	0.025
Uniprot:(Cricetulus barabensis griseus)[10029]	G3IBF4	1	1	35.01	0.02985075
Uniprot:(Cricetulus barabensis griseus)[10029]	G3IBF7	1	1	21.53	0.06
Uniprot:(Cricetulus barabensis griseus)[10029]	G3IBG3	6	9	383.72	0.07372401
Uniprot:(Cricetulus barabensis griseus)[10029]	G3IBH0	3	17	629.41	0.17241379
Uniprot:(Cricetulus barabensis griseus)[10029]	G3IBK2	6	9	292.28	0.02692926
Uniprot:(Cricetulus barabensis griseus)[10029]	G3IBK8	1	1	21.85	0.02494331
Uniprot:(Cricetulus barabensis griseus)[10029]	G3IBK9	1	1	28.06	0.03058824
Uniprot:(Cricetulus barabensis griseus)[10029]	G3IBL4	1	2	44.56	0.01282051
Uniprot:(Cricetulus barabensis griseus)[10029]	G3IBN6	1	1	22.62	0.03745318
Uniprot:(Cricetulus barabensis griseus)[10029]	G3IBN8	1	4	198.94	0.02625821
Uniprot:(Cricetulus barabensis griseus)[10029]	G3IBT1	1	2	45.38	0.01308411
Uniprot:(Cricetulus barabensis griseus)[10029]	G3IBX9	1	1	20.4	0.22222222
Uniprot:(Cricetulus barabensis griseus)[10029]	G3IBY4	2	3	69.2	0.04347826
Uniprot:(Cricetulus barabensis griseus)[10029]	G3IBZ4	2	4	97.05	0.04180064
Uniprot:(Cricetulus barabensis griseus)[10029]	G3IC16	1	1	20.23	0.02272727
Uniprot:(Cricetulus barabensis griseus)[10029]	G3IC31	1	1	23.65	0.06422018
Uniprot:(Cricetulus barabensis griseus)[10029]	G3IC89	1	2	47.25	0.01133145
Uniprot:(Cricetulus barabensis griseus)[10029]	G3IC90	2	4	134.97	0.0356653
Uniprot:(Cricetulus barabensis griseus)[10029]	G3IC99	1	2	51.13	0.02517163
Uniprot:(Cricetulus barabensis griseus)[10029]	G3ICB9	1	1	20.63	0.03399433
Uniprot:(Cricetulus barabensis griseus)[10029]	G3ICC3	1	1	23.44	0.04624278
Uniprot:(Cricetulus barabensis griseus)[10029]	G3ICL7	2	8	288.1	0.11782477
Uniprot:(Cricetulus barabensis griseus)[10029]	G3ICU2	1	1	20.13	0.01659751
Uniprot:(Cricetulus barabensis griseus)[10029]	G3ID19	1	1	21.51	0.04561404
Uniprot:(Cricetulus barabensis griseus)[10029]	G3ID94	1	2	41.99	0.01906158
Uniprot:(Cricetulus barabensis griseus)[10029]	G3IDA9	1	1	23.46	0.01715686
Uniprot:(Cricetulus barabensis griseus)[10029]	G3IDC2	2	6	197.15	0.07594937
Uniprot:(Cricetulus barabensis griseus)[10029]	G3IDD4	8	22	895.96	0.24220624
Uniprot:(Cricetulus barabensis griseus)[10029]	G3IDD7	1	1	22.84	0.03977273
Uniprot:(Cricetulus barabensis griseus)[10029]	G3IDD9	1	1	25.79	0.11111111
Uniprot:(Cricetulus barabensis griseus)[10029]	G3IDJ0	1	1	20.79	0.01565558
Uniprot:(Cricetulus barabensis griseus)[10029]	G3IDL7	4	20	917.52	0.22488038
Uniprot:(Cricetulus barabensis griseus)[10029]	G3IDM2	6	18	622.44	0.59036145
Uniprot:(Cricetulus barabensis griseus)[10029]	G3IDN7	4	10	333.65	0.16740088
Uniprot:(Cricetulus barabensis griseus)[10029]	G3IDS2	1	1	29.12	0.06437768

Uniprot:(Cricetulus barabensis griseus)[10029]	G3IDT6	15	43	1375.1	0.24143302
Uniprot:(Cricetulus barabensis griseus)[10029]	G3IDU7	1	1	26.56	0.01234568
Uniprot:(Cricetulus barabensis griseus)[10029]	G3IE21	2	5	140.71	0.07911392
Uniprot:(Cricetulus barabensis griseus)[10029]	G3IE22	1	2	43.23	0.03249098
Uniprot:(Cricetulus barabensis griseus)[10029]	G3IE55	1	1	22.95	0.05181347
Uniprot:(Cricetulus barabensis griseus)[10029]	G3IEB7	2	7	320	0.125
Uniprot:(Cricetulus barabensis griseus)[10029]	G3IED5	1	3	72.7	0.02244389
Uniprot:(Cricetulus barabensis griseus)[10029]	G3IEE0	1	1	22.65	0.01501502
Uniprot:(Cricetulus barabensis griseus)[10029]	G3IEF1	4	12	480.53	0.25806452
Uniprot:(Cricetulus barabensis griseus)[10029]	G3IEG2	1	2	91.41	0.02645503
Uniprot:(Cricetulus barabensis griseus)[10029]	G3IEK1	2	2	44.31	0.05333333
Uniprot:(Cricetulus barabensis griseus)[10029]	G3IER4	1	1	21.7	0.02071563
Uniprot:(Cricetulus barabensis griseus)[10029]	G3IEU2	4	19	776.53	0.28042328
Uniprot:(Cricetulus barabensis griseus)[10029]	G3IEV7	1	3	67.32	0.03021978
Uniprot:(Cricetulus barabensis griseus)[10029]	G3IEY0	1	1	23.17	0.04932735
Uniprot:(Cricetulus barabensis griseus)[10029]	G3IEY8	1	1	23.77	0.02564103
Uniprot:(Cricetulus barabensis griseus)[10029]	G3IEY9	1	1	42.85	0.07755102
Uniprot:(Cricetulus barabensis griseus)[10029]	G3IF52	7	16	736.96	0.20476191
Uniprot:(Cricetulus barabensis griseus)[10029]	G3IF62	3	6	192.29	0.02746269
Uniprot:(Cricetulus barabensis griseus)[10029]	G3IF80	4	11	294.72	0.07306889
Uniprot:(Cricetulus barabensis griseus)[10029]	G3IFE7	3	4	138.19	0.05806452
Uniprot:(Cricetulus barabensis griseus)[10029]	G3IFJ5	1	1	20.1	0.0546875
Uniprot:(Cricetulus barabensis griseus)[10029]	G3IFJ6	1	4	133.61	0.03719008
Uniprot:(Cricetulus barabensis griseus)[10029]	G3IFR8	1	1	21.44	0.00638421
Uniprot:(Cricetulus barabensis griseus)[10029]	G3IFZ0	2	2	49.44	0.0049505
Uniprot:(Cricetulus barabensis griseus)[10029]	G3IFZ1	1	2	42.82	0.02229299
Uniprot:(Cricetulus barabensis griseus)[10029]	G3IFZ7	1	1	20.8	0.09016393
Uniprot:(Cricetulus barabensis griseus)[10029]	G3IG05	8	16	651.54	0.42213115
Uniprot:(Cricetulus barabensis griseus)[10029]	G3IG06	1	1	23.43	0.01801802
Uniprot:(Cricetulus barabensis griseus)[10029]	G3IG23	1	1	36.39	0.0196281
Uniprot:(Cricetulus barabensis griseus)[10029]	G3IGB7	1	1	27.52	0.01195815
Uniprot:(Cricetulus barabensis griseus)[10029]	G3IGI2	1	1	20.42	0.01146132
Uniprot:(Cricetulus barabensis griseus)[10029]	G3IGL0	1	8	188.61	0.02941177
Uniprot:(Cricetulus barabensis griseus)[10029]	G3IGL3	1	3	75.1	0.01136364
Uniprot:(Cricetulus barabensis griseus)[10029]	G3IGQ2	3	11	445.21	0.44285714
Uniprot:(Cricetulus barabensis griseus)[10029]	G3IGQ3	4	16	603.23	0.23188406
Uniprot:(Cricetulus barabensis griseus)[10029]	G3IGQ4	2	7	190.6	0.17721519
Uniprot:(Cricetulus barabensis griseus)[10029]	G3IGR2	1	1	20.28	0.09411765
Uniprot:(Cricetulus barabensis griseus)[10029]	G3IGS4	1	3	77.49	0.0776699
Uniprot:(Cricetulus barabensis griseus)[10029]	G3IGW0	1	2	63.55	0.09
Uniprot:(Cricetulus barabensis griseus)[10029]	G3IH36	1	2	87.27	0.12631579
Uniprot:(Cricetulus barabensis griseus)[10029]	G3IH63	15	26	1204.4	0.09291581
Uniprot:(Cricetulus barabensis griseus)[10029]	G3IH84	2	5	129.22	0.02895753
Uniprot:(Cricetulus barabensis griseus)[10029]	G3IH96	2	3	73.14	0.02597403
Uniprot:(Cricetulus barabensis griseus)[10029]	G3IHE5	1	1	31.87	0.0462963

Uniprot:(Cricetulus barabensis griseus)[10029]	G3IHH6	3	3	110.16	0.0951087
Uniprot:(Cricetulus barabensis griseus)[10029]	G3IHM2	2	6	137.63	0.04143646
Uniprot:(Cricetulus barabensis griseus)[10029]	G3IHP6	1	1	26.71	0.09160305
Uniprot:(Cricetulus barabensis griseus)[10029]	G3IHR6	1	3	71.76	0.01268116
Uniprot:(Cricetulus barabensis griseus)[10029]	G3IHX3	1	7	160.2	0.04278922
Uniprot:(Cricetulus barabensis griseus)[10029]	G3IHY5	11	41	1393.4	0.25672878
Uniprot:(Cricetulus barabensis griseus)[10029]	G3IHZ9	1	1	20.03	0.05235602
Uniprot:(Cricetulus barabensis griseus)[10029]	G3II00	1	3	70.92	0.01946903
Uniprot:(Cricetulus barabensis griseus)[10029]	G3II58	1	1	20.73	0.03103448
Uniprot:(Cricetulus barabensis griseus)[10029]	G3II91	1	1	20.59	0.00868984
Uniprot:(Cricetulus barabensis griseus)[10029]	G3IIE7	8	30	983.94	0.14112291
Uniprot:(Cricetulus barabensis griseus)[10029]	G3IIF3	2	3	74.78	0.02990033
Uniprot:(Cricetulus barabensis griseus)[10029]	G3IIT8	5	12	400.02	0.12801932
Uniprot:(Cricetulus barabensis griseus)[10029]	G3IIV4	1	1	20.24	0.07723577
Uniprot:(Cricetulus barabensis griseus)[10029]	G3IIZ5	1	1	44.09	0.02644231
Uniprot:(Cricetulus barabensis griseus)[10029]	G3IJ02	1	2	75.07	0.02089137
Uniprot:(Cricetulus barabensis griseus)[10029]	G3IJD6	1	1	20.15	0.05357143
Uniprot:(Cricetulus barabensis griseus)[10029]	G3IJV7	1	1	35.76	0.03738318
Uniprot:(Cricetulus barabensis griseus)[10029]	G3IKC3	3	15	401.08	0.03648649
Uniprot:(Cricetulus barabensis griseus)[10029]	G3IKE5	1	1	25.92	0.01771337
Uniprot:(Cricetulus barabensis griseus)[10029]	G3IKH9	3	10	336.47	0.09708738
Uniprot:(Cricetulus barabensis griseus)[10029]	G3IKI7	1	1	20.39	0.01679389
Uniprot:(Cricetulus barabensis griseus)[10029]	G3IKN5	3	6	220.34	0.10691824
Uniprot:(Cricetulus barabensis griseus)[10029]	G3IKQ5	1	1	36.43	0.03525641
Uniprot:(Cricetulus barabensis griseus)[10029]	G3IKQ6	1	3	148.96	0.12244898
Uniprot:(Cricetulus barabensis griseus)[10029]	G3IKQ9	4	17	473.17	0.33333333
Uniprot:(Cricetulus barabensis griseus)[10029]	G3IKX2	1	2	61.94	0.12765957
Uniprot:(Cricetulus barabensis griseus)[10029]	G3IKZ3	1	1	21.17	0.03651685
Uniprot:(Cricetulus barabensis griseus)[10029]	G3IL75	2	4	94.78	0.0385439
Uniprot:(Cricetulus barabensis griseus)[10029]	G3ILC2	1	2	46.31	0.02733485
Uniprot:(Cricetulus barabensis griseus)[10029]	G3ILF3	3	14	470.45	0.17889908
Uniprot:(Cricetulus barabensis griseus)[10029]	G3ILI8	1	1	31.73	0.03529412
Uniprot:(Cricetulus barabensis griseus)[10029]	G3ILK7	7	19	706.38	0.08173077
Uniprot:(Cricetulus barabensis griseus)[10029]	G3ILN5	5	6	202.31	0.21548822
Uniprot:(Cricetulus barabensis griseus)[10029]	G3IMD1	1	1	22.69	0.02760736
Uniprot:(Cricetulus barabensis griseus)[10029]	G3IMH4	2	7	183.78	0.02866779
Uniprot:(Cricetulus barabensis griseus)[10029]	G3IMR4	1	1	20.27	0.10144928
Uniprot:(Cricetulus barabensis griseus)[10029]	G3IMT1	1	1	21.78	0.00626959
Uniprot:(Cricetulus barabensis griseus)[10029]	G3IMX9	2	5	152.82	0.04715673
Uniprot:(Cricetulus barabensis griseus)[10029]	G3INC5	7	24	1015.7	0.29429429
Uniprot:(Cricetulus barabensis griseus)[10029]	G3INF8	1	1	20.72	0.04761905
Uniprot:(Cricetulus barabensis griseus)[10029]	G3INM4	1	1	20.59	0.05726872
Uniprot:(Cricetulus barabensis griseus)[10029]	G3INR2	1	9	394.71	0.12403101
Uniprot:(Cricetulus barabensis griseus)[10029]	G3INT6	1	2	41.22	0.0345912
Uniprot:(Cricetulus barabensis griseus)[10029]	G3IP52	1	1	20.15	0.04032258

Uniprot:(Cricetulus barabensis griseus)[10029]	G3IPA1	1	1	20.33	0.07462687
Uniprot:(Cricetulus barabensis griseus)[10029]	G3IPD7	3	5	206.11	0.34131737
Uniprot:(Cricetulus barabensis griseus)[10029]	G3IPP8	1	5	199.88	0.25423729
Uniprot:(Cricetulus barabensis griseus)[10029]	G3IPU3	2	4	184.15	0.4
Uniprot:(Cricetulus barabensis griseus)[10029]	G3IQ06	1	1	24.44	0.17241379
Uniprot:(Cricetulus barabensis griseus)[10029]	G3IQ10	1	1	26.62	0.13461539
Uniprot:(Cricetulus barabensis griseus)[10029]	P35950	1	1	22.36	0.02436195
Uniprot:(Cricetulus barabensis griseus)[10029]	Q9EPP7	5	13	408.35	0.14705882
Uniprot:(Cricetulus barabensis griseus)[10029]	X000001	12	1389	63107	0.22234392
Uniprot:(Cricetulus barabensis griseus)[10029]	X000002	23	2787	106557	0.15761162

Supplementary Table S2

Database	AC	# Peptides	# PSMs	Score	% Coverage
Uniprot:(Cricetulus barabensis griseus)[10029]	G3GR67	1	1	20.67	0.01413428
Uniprot:(Cricetulus barabensis griseus)[10029]	G3GRS4	1	1	20.16	0.03204047
Uniprot:(Cricetulus barabensis griseus)[10029]	G3GRW5	1	1	22.86	0.01004184
Uniprot:(Cricetulus barabensis griseus)[10029]	G3GRY4	1	1	26.97	0.02469136
Uniprot:(Cricetulus barabensis griseus)[10029]	G3GSU4	1	6	332.91	0.0931677
Uniprot:(Cricetulus barabensis griseus)[10029]	G3GSZ8	1	1	24.37	0.04901961
Uniprot:(Cricetulus barabensis griseus)[10029]	G3GTB3	1	1	20.82	0.04433498
Uniprot:(Cricetulus barabensis griseus)[10029]	G3GTH6	1	1	22.61	0.07045455
Uniprot:(Cricetulus barabensis griseus)[10029]	G3GTT2	3	13	405.45	0.16783217
Uniprot:(Cricetulus barabensis griseus)[10029]	G3GTX5	1	2	69.04	0.01466276
Uniprot:(Cricetulus barabensis griseus)[10029]	G3GU60	1	1	21.71	0.04278075
Uniprot:(Cricetulus barabensis griseus)[10029]	G3GUU5	1	1	33.8	0.01444623
Uniprot:(Cricetulus barabensis griseus)[10029]	G3GUV4	1	2	63.77	0.00931677
Uniprot:(Cricetulus barabensis griseus)[10029]	G3GVE8	1	1	22.3	0.00668449
Uniprot:(Cricetulus barabensis griseus)[10029]	G3GVK4	1	1	30.79	0.01194217
Uniprot:(Cricetulus barabensis griseus)[10029]	G3GW06	1	1	20.05	0.02196193
Uniprot:(Cricetulus barabensis griseus)[10029]	G3GWA5	1	1	20.42	0.04587156
Uniprot:(Cricetulus barabensis griseus)[10029]	G3GWK6	1	2	49.52	0.00667939
Uniprot:(Cricetulus barabensis griseus)[10029]	G3GXA9	3	6	155.44	0.16304348
Uniprot:(Cricetulus barabensis griseus)[10029]	G3GXB0	2	3	71.24	0.15196078
Uniprot:(Cricetulus barabensis griseus)[10029]	G3GXN3	1	1	26.89	0.00847458
Uniprot:(Cricetulus barabensis griseus)[10029]	G3GXN5	1	1	23.03	0.01315789
Uniprot:(Cricetulus barabensis griseus)[10029]	G3GXZ0	5	14	429.32	0.0845481
Uniprot:(Cricetulus barabensis griseus)[10029]	G3GYE3	1	2	43.81	0.00658514
Uniprot:(Cricetulus barabensis griseus)[10029]	G3GYP9	6	36	1214	0.32160804
Uniprot:(Cricetulus barabensis griseus)[10029]	G3GZ40	1	1	22.41	0.00362506
Uniprot:(Cricetulus barabensis griseus)[10029]	G3GZG1	1	1	20.98	0.01129944
Uniprot:(Cricetulus barabensis griseus)[10029]	G3H0C9	1	2	70.91	0.07051282
Uniprot:(Cricetulus barabensis griseus)[10029]	G3H0L9	2	6	231.77	0.07964602
Uniprot:(Cricetulus barabensis griseus)[10029]	G3H0R3	1	1	32.74	0.01593625
Uniprot:(Cricetulus barabensis griseus)[10029]	G3H0S7	1	1	32.38	0.24074074
Uniprot:(Cricetulus barabensis griseus)[10029]	G3H0U6	6	16	447.48	0.16441441
Uniprot:(Cricetulus barabensis griseus)[10029]	G3H1J6	1	1	24.04	0.01129032
Uniprot:(Cricetulus barabensis griseus)[10029]	G3H2D8	1	4	108.65	0.14529915
Uniprot:(Cricetulus barabensis griseus)[10029]	G3H2H4	1	1	26.19	0.00870827
Uniprot:(Cricetulus barabensis griseus)[10029]	G3H2L3	1	1	20.53	0.02819237
Uniprot:(Cricetulus barabensis griseus)[10029]	G3H2T8	1	2	66.38	0.06923077
Uniprot:(Cricetulus barabensis griseus)[10029]	G3H354	1	1	37.85	0.02710843
Uniprot:(Cricetulus barabensis griseus)[10029]	G3H3G3	1	1	21.85	0.01236094
Uniprot:(Cricetulus barabensis griseus)[10029]	G3H3H1	1	1	20.52	0.01328021
Uniprot:(Cricetulus barabensis griseus)[10029]	G3H3N8	1	1	20.45	0.01996672
Uniprot:(Cricetulus barabensis griseus)[10029]	G3H3Q1	6	11	336.27	0.22669492
Uniprot:(Cricetulus barabensis griseus)[10029]	G3H3X1	1	2	43.44	0.02124183
Uniprot:(Cricetulus barabensis griseus)[10029]	G3H4I2	7	15	537.85	0.22641509

Uniprot:(Cricetulus barabensis griseus)[10029]	G3H4Z8	2	2	47.13	0.03943218
Uniprot:(Cricetulus barabensis griseus)[10029]	G3H533	1	5	202.48	0.06018519
Uniprot:(Cricetulus barabensis griseus)[10029]	G3H577	1	1	20.14	0.05235602
Uniprot:(Cricetulus barabensis griseus)[10029]	G3H584	1	1	28.9	0.0562249
Uniprot:(Cricetulus barabensis griseus)[10029]	G3H5D5	1	1	44.16	0.13445378
Uniprot:(Cricetulus barabensis griseus)[10029]	G3H6G8	1	1	21.44	0.05641026
Uniprot:(Cricetulus barabensis griseus)[10029]	G3H6I5	1	2	43.01	0.02114804
Uniprot:(Cricetulus barabensis griseus)[10029]	G3H6Y5	1	1	28.49	0.17721519
Uniprot:(Cricetulus barabensis griseus)[10029]	G3H6Z2	1	1	22.43	0.07666667
Uniprot:(Cricetulus barabensis griseus)[10029]	G3H705	1	2	47.95	0.11764706
Uniprot:(Cricetulus barabensis griseus)[10029]	G3H7B3	4	10	306.65	0.17437722
Uniprot:(Cricetulus barabensis griseus)[10029]	G3H7D7	1	1	24.91	0.06214689
Uniprot:(Cricetulus barabensis griseus)[10029]	G3H7F7	1	1	20.93	0.08333333
Uniprot:(Cricetulus barabensis griseus)[10029]	G3H7R5	1	1	24.86	0.10294118
Uniprot:(Cricetulus barabensis griseus)[10029]	G3H8E3	1	1	35.36	0.03278689
Uniprot:(Cricetulus barabensis griseus)[10029]	G3H8F4	1	2	57.26	0.01791713
Uniprot:(Cricetulus barabensis griseus)[10029]	G3H8W0	1	1	25.88	0.13157895
Uniprot:(Cricetulus barabensis griseus)[10029]	G3H8X7	1	1	21.18	0.00296638
Uniprot:(Cricetulus barabensis griseus)[10029]	G3H9P0	1	4	94.85	0.05714286
Uniprot:(Cricetulus barabensis griseus)[10029]	G3H9R7	1	1	23.45	0.06926407
Uniprot:(Cricetulus barabensis griseus)[10029]	G3HA23	1	1	20.25	0.05166052
Uniprot:(Cricetulus barabensis griseus)[10029]	G3HA54	1	1	23.31	0.02962963
Uniprot:(Cricetulus barabensis griseus)[10029]	G3HAI1	1	1	20.36	0.0131291
Uniprot:(Cricetulus barabensis griseus)[10029]	G3HB04	4	5	170.28	0.18532819
Uniprot:(Cricetulus barabensis griseus)[10029]	G3HB78	1	2	41.13	0.02325581
Uniprot:(Cricetulus barabensis griseus)[10029]	G3HB87	1	2	48.8	0.0053286
Uniprot:(Cricetulus barabensis griseus)[10029]	G3HBD3	3	6	155.5	0.19078947
Uniprot:(Cricetulus barabensis griseus)[10029]	G3HBG8	1	1	37.68	0.13664596
Uniprot:(Cricetulus barabensis griseus)[10029]	G3HBH2	1	1	22.11	0.03821656
Uniprot:(Cricetulus barabensis griseus)[10029]	G3HBL5	1	1	23.15	0.01856148
Uniprot:(Cricetulus barabensis griseus)[10029]	G3HBV4	1	1	20.89	0.01859504
Uniprot:(Cricetulus barabensis griseus)[10029]	G3HBX3	1	1	20.66	0.00538048
Uniprot:(Cricetulus barabensis griseus)[10029]	G3HC25	3	9	272.56	0.31632653
Uniprot:(Cricetulus barabensis griseus)[10029]	G3HC29	1	1	21.47	0.06930693
Uniprot:(Cricetulus barabensis griseus)[10029]	G3HC31	4	18	485.61	0.34831461
Uniprot:(Cricetulus barabensis griseus)[10029]	G3HCB8	1	2	40.6	0.00479721
Uniprot:(Cricetulus barabensis griseus)[10029]	G3HCI1	1	1	24.34	0.01863354
Uniprot:(Cricetulus barabensis griseus)[10029]	G3HCW9	3	4	139.71	0.18181818
Uniprot:(Cricetulus barabensis griseus)[10029]	G3HCX8	5	7	183.7	0.10071942
Uniprot:(Cricetulus barabensis griseus)[10029]	G3HD30	1	1	20.7	0.00647668
Uniprot:(Cricetulus barabensis griseus)[10029]	G3HDU9	1	1	26.49	0.03755869
Uniprot:(Cricetulus barabensis griseus)[10029]	G3HFQ5	1	1	22.2	0.01392111
Uniprot:(Cricetulus barabensis griseus)[10029]	G3HFW4	1	1	23.58	0.01570681
Uniprot:(Cricetulus barabensis griseus)[10029]	G3HGL5	1	1	21.39	0.01052632
Uniprot:(Cricetulus barabensis griseus)[10029]	G3HH30	1	2	50.98	0.03481013

Uniprot:(Cricetulus barabensis griseus)[10029]	G3HH31	1	1	20.11	0.00990099
Uniprot:(Cricetulus barabensis griseus)[10029]	G3HH47	1	3	98.4	0.125
Uniprot:(Cricetulus barabensis griseus)[10029]	G3HH86	1	1	21.75	0.04056795
Uniprot:(Cricetulus barabensis griseus)[10029]	G3HHM5	1	1	24.05	0.00143
Uniprot:(Cricetulus barabensis griseus)[10029]	G3HHN5	1	1	20.37	0.03102625
Uniprot:(Cricetulus barabensis griseus)[10029]	G3HHR3	1	2	67.44	0.027897
Uniprot:(Cricetulus barabensis griseus)[10029]	G3HI77	1	1	21.14	0.00804598
Uniprot:(Cricetulus barabensis griseus)[10029]	G3HJ36	1	1	27.97	0.04347826
Uniprot:(Cricetulus barabensis griseus)[10029]	G3HJ82	1	1	23.14	0.01818182
Uniprot:(Cricetulus barabensis griseus)[10029]	G3HJ99	1	1	22.98	0.00574713
Uniprot:(Cricetulus barabensis griseus)[10029]	G3HJS3	1	1	24.04	0.00582751
Uniprot:(Cricetulus barabensis griseus)[10029]	G3HJT5	1	1	27.14	0.04281346
Uniprot:(Cricetulus barabensis griseus)[10029]	G3HLK9	1	1	28.87	0.02134146
Uniprot:(Cricetulus barabensis griseus)[10029]	G3HLS2	1	1	27.3	0.03688525
Uniprot:(Cricetulus barabensis griseus)[10029]	G3HLY2	1	1	22.29	0.02143758
Uniprot:(Cricetulus barabensis griseus)[10029]	G3HM99	1	5	195.51	0.03952569
Uniprot:(Cricetulus barabensis griseus)[10029]	G3HMB8	1	3	75.79	0.06034483
Uniprot:(Cricetulus barabensis griseus)[10029]	G3HMG4	2	2	71.45	0.04387991
Uniprot:(Cricetulus barabensis griseus)[10029]	G3HNR1	1	1	20.66	0.00825472
Uniprot:(Cricetulus barabensis griseus)[10029]	G3HNV4	1	1	22.49	0.08666667
Uniprot:(Cricetulus barabensis griseus)[10029]	G3HNY5	1	1	27.25	0.03305785
Uniprot:(Cricetulus barabensis griseus)[10029]	G3HP72	1	1	20.8	0.0112782
Uniprot:(Cricetulus barabensis griseus)[10029]	G3HPE1	1	1	22.25	0.11320755
Uniprot:(Cricetulus barabensis griseus)[10029]	G3HQM6	5	5	136.21	0.06475716
Uniprot:(Cricetulus barabensis griseus)[10029]	G3HRU5	1	3	61.28	0.01040462
Uniprot:(Cricetulus barabensis griseus)[10029]	G3HS91	1	2	44.52	0.00790514
Uniprot:(Cricetulus barabensis griseus)[10029]	G3HSG3	1	1	20.52	0.00499546
Uniprot:(Cricetulus barabensis griseus)[10029]	G3HST3	1	1	23.56	0.04982206
Uniprot:(Cricetulus barabensis griseus)[10029]	G3HSW2	1	8	176.12	0.05240175
Uniprot:(Cricetulus barabensis griseus)[10029]	G3HSX8	2	9	250.25	0.04065041
Uniprot:(Cricetulus barabensis griseus)[10029]	G3HSY4	1	1	22.6	0.03378378
Uniprot:(Cricetulus barabensis griseus)[10029]	G3HT52	1	1	22.35	0.13432836
Uniprot:(Cricetulus barabensis griseus)[10029]	G3HTG4	1	1	21.01	0.00961538
Uniprot:(Cricetulus barabensis griseus)[10029]	G3HTI8	1	1	21.49	0.02549575
Uniprot:(Cricetulus barabensis griseus)[10029]	G3HTZ1	1	6	135.44	0.01372998
Uniprot:(Cricetulus barabensis griseus)[10029]	G3HTZ9	1	1	20.45	0.03338392
Uniprot:(Cricetulus barabensis griseus)[10029]	G3HUR8	1	1	20.42	0.02953586
Uniprot:(Cricetulus barabensis griseus)[10029]	G3HV18	1	1	22	0.13432836
Uniprot:(Cricetulus barabensis griseus)[10029]	G3HVB6	1	1	24.74	0.00830816
Uniprot:(Cricetulus barabensis griseus)[10029]	G3HVI7	1	1	20.12	0.00525486
Uniprot:(Cricetulus barabensis griseus)[10029]	G3HVK4	1	1	23.2	0.02535658
Uniprot:(Cricetulus barabensis griseus)[10029]	G3HWE6	1	2	45.93	0.01458886
Uniprot:(Cricetulus barabensis griseus)[10029]	G3HWE7	2	2	99.55	0.08908046
Uniprot:(Cricetulus barabensis griseus)[10029]	G3HWQ8	1	1	20.41	0.03529412
Uniprot:(Cricetulus barabensis griseus)[10029]	G3HWY2	1	1	22.13	0.01854494

Uniprot:(Cricetulus barabensis griseus)[10029]	G3HX05	1	2	49.12	0.02108434
Uniprot:(Cricetulus barabensis griseus)[10029]	G3HX39	1	1	29.22	0.04102564
Uniprot:(Cricetulus barabensis griseus)[10029]	G3HX97	1	1	22.54	0.10555556
Uniprot:(Cricetulus barabensis griseus)[10029]	G3HXF7	1	1	30.59	0.02622951
Uniprot:(Cricetulus barabensis griseus)[10029]	G3HXH6	1	1	20.54	0.06451613
Uniprot:(Cricetulus barabensis griseus)[10029]	G3HYT3	1	1	21.46	0.00676437
Uniprot:(Cricetulus barabensis griseus)[10029]	G3HZV5	1	1	21.2	0.03565062
Uniprot:(Cricetulus barabensis griseus)[10029]	G3I0W1	1	8	245.26	0.07194245
Uniprot:(Cricetulus barabensis griseus)[10029]	G3I1H5	1	2	78.4	0.02511416
Uniprot:(Cricetulus barabensis griseus)[10029]	G3I1P5	3	7	226.15	0.28070175
Uniprot:(Cricetulus barabensis griseus)[10029]	G3I216	2	3	83.83	0.12299465
Uniprot:(Cricetulus barabensis griseus)[10029]	G3I225	1	1	25.54	0.01891253
Uniprot:(Cricetulus barabensis griseus)[10029]	G3I255	3	8	232.19	0.06806283
Uniprot:(Cricetulus barabensis griseus)[10029]	G3I278	1	1	20.19	0.00556586
Uniprot:(Cricetulus barabensis griseus)[10029]	G3I295	1	5	117.76	0.00632911
Uniprot:(Cricetulus barabensis griseus)[10029]	G3I2T9	1	1	28.09	0.01627034
Uniprot:(Cricetulus barabensis griseus)[10029]	G3I2U0	1	1	25.73	0.11940299
Uniprot:(Cricetulus barabensis griseus)[10029]	G3I3D7	1	2	43.15	0.01311475
Uniprot:(Cricetulus barabensis griseus)[10029]	G3I3X4	6	21	783.27	0.1510989
Uniprot:(Cricetulus barabensis griseus)[10029]	G3I3Y6	1	1	53.04	0.07239819
Uniprot:(Cricetulus barabensis griseus)[10029]	G3I4H6	5	13	412.03	0.21153846
Uniprot:(Cricetulus barabensis griseus)[10029]	G3I4Z7	1	6	172.73	0.05925926
Uniprot:(Cricetulus barabensis griseus)[10029]	G3I5K8	1	1	22.04	0.03125
Uniprot:(Cricetulus barabensis griseus)[10029]	G3I6C6	1	1	21.05	0.0887574
Uniprot:(Cricetulus barabensis griseus)[10029]	G3I6P1	1	1	23.93	0.06956522
Uniprot:(Cricetulus barabensis griseus)[10029]	G3I7K4	1	1	32.74	0.04545455
Uniprot:(Cricetulus barabensis griseus)[10029]	G3I8C8	1	1	23.72	0.0244898
Uniprot:(Cricetulus barabensis griseus)[10029]	G3I8R9	16	39	1314.5	0.30122324
Uniprot:(Cricetulus barabensis griseus)[10029]	G3I936	2	2	47.62	0.15436242
Uniprot:(Cricetulus barabensis griseus)[10029]	G3I948	1	2	53.05	0.05228758
Uniprot:(Cricetulus barabensis griseus)[10029]	G3I952	3	5	183.87	0.3381295
Uniprot:(Cricetulus barabensis griseus)[10029]	G3I990	1	2	41.32	0.05076142
Uniprot:(Cricetulus barabensis griseus)[10029]	G3I9K9	1	1	25.52	0.00377766
Uniprot:(Cricetulus barabensis griseus)[10029]	G3I9L9	1	1	20.84	0.00414938
Uniprot:(Cricetulus barabensis griseus)[10029]	G3I9P6	1	1	22.77	0.02886598
Uniprot:(Cricetulus barabensis griseus)[10029]	G3I9T4	1	1	22.38	0.10344828
Uniprot:(Cricetulus barabensis griseus)[10029]	G3IA08	1	5	128.45	0.02325581
Uniprot:(Cricetulus barabensis griseus)[10029]	G3IA91	1	1	26.37	0.05323194
Uniprot:(Cricetulus barabensis griseus)[10029]	G3IAG8	1	1	29.4	0.0120614
Uniprot:(Cricetulus barabensis griseus)[10029]	G3IAQ0	9	39	1159.3	0.2983683
Uniprot:(Cricetulus barabensis griseus)[10029]	G3IAX0	1	1	27.68	0.01026393
Uniprot:(Cricetulus barabensis griseus)[10029]	G3IAX3	6	19	712.34	0.14750542
Uniprot:(Cricetulus barabensis griseus)[10029]	G3IAX5	1	1	21.81	0.04878049
Uniprot:(Cricetulus barabensis griseus)[10029]	G3IBH0	1	13	482.59	0.0591133
Uniprot:(Cricetulus barabensis griseus)[10029]	G3IBN8	1	6	297.98	0.02625821

Uniprot:(Cricetulus barabensis griseus)[10029]	G3IC13	1	1	25.65	0.01014493
Uniprot:(Cricetulus barabensis griseus)[10029]	G3IC22	1	1	23.87	0.01086957
Uniprot:(Cricetulus barabensis griseus)[10029]	G3ICC3	1	1	21.43	0.05780347
Uniprot:(Cricetulus barabensis griseus)[10029]	G3ID60	1	1	28.42	0.32692308
Uniprot:(Cricetulus barabensis griseus)[10029]	G3ID67	1	2	43.78	0.05617978
Uniprot:(Cricetulus barabensis griseus)[10029]	G3IDC2	1	4	109.47	0.02953586
Uniprot:(Cricetulus barabensis griseus)[10029]	G3IDD4	2	3	97.2	0.0383693
Uniprot:(Cricetulus barabensis griseus)[10029]	G3IDE2	1	2	52.49	0.03977273
Uniprot:(Cricetulus barabensis griseus)[10029]	G3IDM2	1	4	112.51	0.06626506
Uniprot:(Cricetulus barabensis griseus)[10029]	G3IDT6	3	3	83.65	0.03894081
Uniprot:(Cricetulus barabensis griseus)[10029]	G3IEF1	2	6	190.16	0.11290323
Uniprot:(Cricetulus barabensis griseus)[10029]	G3IEU2	1	3	120.96	0.07936508
Uniprot:(Cricetulus barabensis griseus)[10029]	G3IEV7	1	1	23.27	0.03021978
Uniprot:(Cricetulus barabensis griseus)[10029]	G3IF52	2	2	47.44	0.03809524
Uniprot:(Cricetulus barabensis griseus)[10029]	G3IF79	1	1	23.41	0.00354072
Uniprot:(Cricetulus barabensis griseus)[10029]	G3IFB0	1	1	25.31	0.00752418
Uniprot:(Cricetulus barabensis griseus)[10029]	G3IFQ1	1	2	45	0.00875912
Uniprot:(Cricetulus barabensis griseus)[10029]	G3IFX3	1	3	65.21	0.02352941
Uniprot:(Cricetulus barabensis griseus)[10029]	G3IGC5	1	1	23.57	0.02445652
Uniprot:(Cricetulus barabensis griseus)[10029]	G3IGH6	1	1	20.77	0.01217391
Uniprot:(Cricetulus barabensis griseus)[10029]	G3IGQ2	2	4	110.57	0.28571429
Uniprot:(Cricetulus barabensis griseus)[10029]	G3IGQ3	1	1	35.66	0.06763285
Uniprot:(Cricetulus barabensis griseus)[10029]	G3IHV0	1	1	20.92	0.03478261
Uniprot:(Cricetulus barabensis griseus)[10029]	G3IHY5	2	3	157.08	0.04968944
Uniprot:(Cricetulus barabensis griseus)[10029]	G3IK42	1	1	20.57	0.28
Uniprot:(Cricetulus barabensis griseus)[10029]	G3IK80	1	1	20.19	0.07627119
Uniprot:(Cricetulus barabensis griseus)[10029]	G3IKC3	1	2	84.26	0.01621622
Uniprot:(Cricetulus barabensis griseus)[10029]	G3IKD7	1	3	96.97	0.152
Uniprot:(Cricetulus barabensis griseus)[10029]	G3IKQ6	1	1	21.19	0.07142857
Uniprot:(Cricetulus barabensis griseus)[10029]	G3IKQ9	4	12	365.94	0.4017094
Uniprot:(Cricetulus barabensis griseus)[10029]	G3IKR5	1	1	24.09	0.01030928
Uniprot:(Cricetulus barabensis griseus)[10029]	G3ILS9	1	1	20.68	0.00801603
Uniprot:(Cricetulus barabensis griseus)[10029]	G3IN97	1	1	20.93	0.10909091
Uniprot:(Cricetulus barabensis griseus)[10029]	G3INC5	7	10	348.35	0.32432432
Uniprot:(Cricetulus barabensis griseus)[10029]	G3INL0	1	1	21.36	0.03846154
Uniprot:(Cricetulus barabensis griseus)[10029]	G3IP80	1	1	20.86	0.04878049
Uniprot:(Cricetulus barabensis griseus)[10029]	G3IPU3	1	1	24.03	0.15714286
Uniprot:(Cricetulus barabensis griseus)[10029]	Q9EPP7	4	11	348.49	0.11437908
Uniprot:(Cricetulus barabensis griseus)[10029]	X000001	14	1322	54700	0.28696605
Uniprot:(Cricetulus barabensis griseus)[10029]	X000002	29	2171	80706	0.19795589

Supplementary Table S3. Relative ratios of the heavy and light mAb chains during DSP. The mean ratio intensity values of the data are presented, calculated relative Protein A Eluate replicate 1, to which a ratio value of 1 was assigned.

**Samples**	**Heavy:Light chain ratio**
HCCF 1	0.50
HCCF 2	0.50
Protein A Eluate 1	1.00
Protein A Eluate 2	0.95
Cation Exchange Eluate 1	0.89
Cation Exchange Eluate 2	0.87
Anion Exchange Flow Through 1	0.85
Anion Exchange Flow Through 2	0.81

Supplementary Table S4

Above LOD (3+ unique peptides)

iTRAQ 1 HCCF	iTRAQ 1 FT	iTRAQ 1 Prot A	iTRAQ 2 HCCF	iTRAQ 2 Prot A	iTRAQ 2 CatX	iTRAQ 2 AnX
G3GR64	G3H0U6	G3H0U6	G3GTT2	X000002	X000002	X000002
G3GR73	G3HC84	G3HB04	G3GXA9	G3GYP9	X000001	G3GYP9
G3GS70	G3GXB0	G3HSE4	G3GXZ0	G3HC31		X000001
G3GSG4	G3HB04	G3HKZ1	G3GYP9	X000001		
G3GTC7	G3HGM6	G3HNJ3	G3H0U6	G3I3X4		
G3GTT2	G3H8V1	G3HSX8	G3H3Q1	G3IAX3		
G3GTX5	G3HSE4	G3HQL6	G3H4I2	Q9EPP7		
G3GU60	G3HKZ1	G3IIE7	G3H7B3	G3I8R9		
G3GUU5	G3HNJ3	G3IAQ0	G3HB04			
G3GUV4	G3HSX8	G3IBG3	G3HBD3			
G3GVD0	G3HQL6	G3H0S7	G3HC25			
G3GVX1	G3HSM3	G3GXZ0	G3HC31			
G3GVX2	G3H935	G3HQY2	G3HCW9			
G3GWB3	G3IIE7	G3ILK7	G3HCX8			
G3GWQ1	G3IAQ0	G3IIT8	G3HQM6			
G3GWR8	G3IBG3	X000002	G3I1P5			
G3GXA9	G3HGP4	G3GYP9	G3I255			
G3GXB0	G3I9F0	G3H3Q1	G3I3X4			
G3GXD7	G3HTE5	G3IF52	G3I4H6			
G3GXS2	G3HAP7	G3HWC3	G3I8R9			
G3GXZ0	G3H0S7	G3HG95	G3I952			
G3GY17	G3H0S4	G3HIM1	G3IAQ0			
G3GYP7	G3GXZ0	G3H0E4	G3IAX3			
G3GYP9	G3I6T1	G3HH30	G3IDT6			
G3GZ90	G3HQY2	G3ILN5	G3IKQ9			
G3GZB2	G3I1V3	G3HSL4	G3INC5			
G3GZD2	G3HG83	G3I877	Q9EPP7			
G3GZW8	G3ILK7	G3HMV7	X000001			
G3GZZ0	G3HU10	G3H0L9	X000002			
G3H0C2	G3IIT8	G3GR64				
G3H0C9	G3HYB7	X000001				
G3H0E4	G3I5Z5	G3I3X4				
G3H0L9	G3HZ42	G3I4E8				
G3H0S4	G3IKQ9	G3GR73				

G3H0S7	G3IDN7	G3GWQ1
G3H0U6	X000002	G3GWR8
G3H1K9	G3GYP9	G3HMG4
G3H2C4	G3H3Q1	G3GXD7
G3H2K2	G3IF52	G3HQM6
G3H354	G3HWC3	Q9EPP7
G3H3D3	G3HG95	G3HN14
G3H3E4	G3HIM1	G3IDT6
G3H3Q1	G3IFE7	G3H354
G3H4I2	G3HA54	G3HFM4
G3H4T5	G3H0E4	G3HCX8
G3H4V1	G3HLV6	G3HWJ3
G3H4Z8	G3HKG9	G3I8R9
G3H533	G3I3G8	G3GZB2
G3H577	G3HC31	G3HNT9
G3H584	G3HR08	G3IAX3
G3H5V0	G3HH30	G3GUU5
G3H5W0	G3IBK2	G3IBH0
G3H697	G3H4T5	G3H4I2
G3H6I5	G3H8Y4	G3HGY8
G3H6T5	G3HYJ9	G3HX39
G3H705	G3ILN5	G3H1K9
G3H7B3	G3HSL4	G3I255
G3H7Z2	G3HSF3	G3I3Y6
G3H8F4	G3GYP7	G3H8V5
G3H8V1	G3H8V4	G3HC25
G3H8V4	G3I877	G3HC29
G3H8V5	G3H0C2	G3HRK9
G3H8Y4	G3HMV7	G3IEU2
G3H8Y5	G3H0L9	G3HHR3
G3H928	G3H8F4	G3I1Y9
G3H935	G3GR64	G3IGQ3
G3HA54	G3H6T5	G3GTC7
G3HAP7	X000001	G3GTT2
G3HB04	G3GXS2	G3IDD4
G3HBD3	G3I3X4	G3I4H6
G3HBD4	G3H577	G3H584

G3HBI9	G3I4E8	G3IG05
G3HC25	G3GR73	G3HBD3
G3HC29	G3GWQ1	G3HBD4
G3HC31	G3GWR8	G3IH63
G3HC84	G3HMG4	G3IEF1
G3HCW9	G3GXD7	G3GUV4
G3HCX8	G3HQM6	G3I278
G3HD97	G3GZ90	G3H2K2
G3HDQ1	Q9EPP7	G3IDM2
G3HDQ2	G3GTX5	G3I1H5
G3HDT6	G3HXF7	G3INC5
G3HFM4	G3HMA1	G3HU51
G3HG83	G3HN14	G3I9G7
G3HG95	G3IDT6	G3GZZ0
G3HGM6	G3H354	G3H3E4
G3HGP4	G3HDQ2	
G3HGY8	G3HFM4	
G3HH02	G3I952	
G3HH30	G3HCX8	
G3HHR3	G3HWJ3	
G3HIM1	G3HRK0	
G3HK90	G3GXA9	
G3HKG9	G3I8R9	
G3HKQ7	G3IGQ2	
G3HKZ1	G3GZB2	
G3HLS2	G3GSG4	
G3HLT3	G3HNT9	
G3HLV6	G3HLS2	
G3HM03	G3IHY5	
G3HMA1	G3IAX3	
G3HMG4	G3GUU5	
G3HMQ0	G3ILF3	
G3HMV7	G3H533	
G3HN14	G3I5L3	
G3HN88	G3IBH0	
G3HNJ3	G3H4I2	
G3HNT9	G3IF62	

G3HNY5	G3HGY8
G3HQL6	G3HX39
G3HQM6	G3H1K9
G3HQP8	G3I255
G3HQY2	G3I3Y6
G3HR08	G3H8V5
G3HRK0	G3HC25
G3HRK9	G3HC29
G3HSE4	G3H6I5
G3HSF3	G3HRK9
G3HSL4	G3HMQ0
G3HSM3	G3H4V1
G3HSX8	G3I129
G3HTE5	G3H4Z8
G3HTG9	G3H8Y5
G3HU10	G3IEU2
G3HU28	G3GVX1
G3HU51	G3GZD2
G3HV83	G3HHR3
G3HW06	G3I1Y9
G3HWC3	G3IGQ3
G3HWE4	G3IAI6
G3HWE7	G3I4D4
G3HWJ3	G3HTG9
G3HX39	G3HBI9
G3HXF7	G3I064
G3HYB7	G3HV83
G3HYJ9	G3H705
G3HZ42	G3GTC7
G3I015	G3GTT2
G3I064	G3HH02
G3I129	G3IDD4
G3I1H5	G3H5V0
G3I1P5	G3I4H6
G3I1V3	G3H584
G3I1Y9	G3I8P7
G3I255	G3I015

G3I278	G3IG05
G3I2H0	G3HBD3
G3I2K6	G3I4W7
G3I2M1	G3GVD0
G3I3G8	G3HBD4
G3I3H2	G3I973
G3I3U5	G3IH63
G3I3X4	G3GY17
G3I3Y6	G3IF80
G3I4D4	G3HDQ1
G3I4E8	G3HDT6
G3I4H6	G3GS70
G3I4W7	G3IEF1
G3I5L3	G3GUV4
G3I5T9	G3I278
G3I5Z5	G3H2K2
G3I6T1	G3H697
G3I877	G3HCW9
G3I8P7	G3IHH6
G3I8R9	G3GU60
G3I952	G3I1P5
G3I973	G3HKQ7
G3I9F0	G3H5W0
G3I9G7	G3GWB3
G3IAI6	G3IDL7
G3IAQ0	G3HU28
G3IAX3	G3I3U5
G3IBG3	G3H7Z2
G3IBH0	G3HWE4
G3IBK2	G3HK90
G3IDD4	G3IKC3
G3IDL7	G3I2M1
G3IDM2	G3IDM2
G3IDN7	G3HNY5
G3IDT6	G3I1H5
G3IEF1	G3HD97
G3IEU2	G3I2K6

G3IF52	G3IKN5
G3IF62	G3IPD7
G3IF80	G3HM03
G3IFE7	G3I5T9
G3IG05	G3HW06
G3IGQ2	G3H928
G3IGQ3	G3H7B3
G3IH63	G3INC5
G3IHH6	G3I2H0
G3IHY5	G3H0C9
G3IIE7	G3IKH9
G3IIT8	G3GVX2
G3IKC3	G3H2C4
G3IKH9	G3HU51
G3IKN5	G3I9G7
G3IKQ9	G3I3H2
G3ILF3	G3HN88
G3ILK7	G3H3D3
G3ILN5	G3HLT3
G3INC5	G3HWE7
G3IPD7	G3GZZ0
Q9EPP7	G3HQP8
X000001	G3H3E4
X000002	G3GZW8

Preferentially retained

iTRAQ 1 HCCF vs FT	iTRAQ 1 Prot A vs HCCF
G3GU60	G3I9G7
G3GXS2	G3IBG3
G3H8Y4	
G3HQP8	
G3HU10	
G3IBG3	
G3IF62	
G3IF80	

Decreased in Level

iTRAQ 1 HCCF vs FT	iTRAQ 1 Prot A vs HCCF	iTRAQ 2 Prot A	iTRAQ 2 CatX	iTRAQ 2 AnX
G3GR64	G3GR73	G3GTT2	G3GTT2	G3GTT2
G3GR73	G3GS70	G3GXA9	G3GXA9	G3GXA9
G3GTC7	G3GSG4	G3GXZ0	G3GXZ0	G3GXZ0
G3GTT2	G3GTC7	G3GYP9	G3GYP9	G3GYP9
G3GTX5	G3GTT2	G3H0U6	G3H0U6	G3H0U6
G3GUU5	G3GTX5	G3H3Q1	G3H3Q1	G3H3Q1
G3GUV4	G3GU60	G3H4I2	G3H4I2	G3H4I2
G3GVD0	G3GUU5	G3H7B3	G3H7B3	G3H7B3
G3GVX1	G3GUV4	G3HB04	G3HB04	G3HB04
G3GVX2	G3GVD0	G3HBD3	G3HBD3	G3HBD3
G3GWQ1	G3GVX1	G3HC25	G3HC25	G3HC25
G3GWR8	G3GVX2	G3HC31	G3HC31	G3HC31
G3GXA9	G3GWB3	G3HCW9	G3HCW9	G3HCW9
G3GXB0	G3GWQ1	G3HCX8	G3HCX8	G3HCX8
G3GXZ0	G3GWR8	G3HQM6	G3HQM6	G3HQM6
G3GYP7	G3GXA9	G3I1P5	G3I1P5	G3I1P5
G3GYP9	G3GXB0	G3I255	G3I255	G3I255
G3GZ90	G3GXS2	G3I3X4	G3I3X4	G3I3X4
G3GZB2	G3GXZ0	G3I4H6	G3I4H6	G3I4H6
G3GZD2	G3GY17	G3I8R9	G3I8R9	G3I8R9
G3GZW8	G3GYP7	G3I952	G3I952	G3I952
G3GZZ0	G3GYP9	G3IAQ0	G3IAQ0	G3IAQ0
G3H0C2	G3GZ90	G3IAX3	G3IAX3	G3IAX3
G3H0C9	G3GZB2	G3IDT6	G3IDT6	G3IDT6
G3H0E4	G3GZD2	G3IKQ9	G3IKQ9	G3IKQ9
G3H0L9	G3GZW8	G3INC5	G3INC5	G3INC5
G3H0S4	G3GZZ0	Q9EPP7	Q9EPP7	Q9EPP7
G3H0S7	G3H0C2			
G3H0U6	G3H0C9			
G3H1K9	G3H0E4			
G3H2C4	G3H0L9			
G3H354	G3H0S4			
G3H3Q1	G3H0S7			
G3H4I2	G3H0U6			
G3H4V1	G3H2C4			

G3H4Z8	G3H354
G3H533	G3H3D3
G3H577	G3H3Q1
G3H584	G3H4I2
G3H697	G3H4T5
G3H6I5	G3H4V1
G3H6T5	G3H4Z8
G3H705	G3H533
G3H7B3	G3H577
G3H8F4	G3H584
G3H8V1	G3H5V0
G3H8V4	G3H5W0
G3H8V5	G3H697
G3H8Y5	G3H6I5
G3H928	G3H6T5
G3H935	G3H705
G3HA54	G3H7B3
G3HAP7	G3H7Z2
G3HB04	G3H8F4
G3HBD3	G3H8V1
G3HBD4	G3H8V4
G3HBI9	G3H8V5
G3HC25	G3H8Y4
G3HC29	G3H8Y5
G3HC31	G3H928
G3HC84	G3H935
G3HCW9	G3HA54
G3HCX8	G3HAP7
G3HD97	G3HB04
G3HDQ1	G3HBD3
G3HDQ2	G3HBD4
G3HFM4	G3HBI9
G3HG83	G3HC25
G3HG95	G3HC29
G3HGM6	G3HC31
G3HGP4	G3HC84
G3HGY8	G3HCW9

G3HH02	G3HCX8
G3HH30	G3HD97
G3HHR3	G3HDQ1
G3HIM1	G3HDQ2
G3HK90	G3HDT6
G3HKQ7	G3HFM4
G3HKZ1	G3HG83
G3HLS2	G3HG95
G3HLT3	G3HGM6
G3HLV6	G3HGP4
G3HMA1	G3HGY8
G3HMG4	G3HH02
G3HMQ0	G3HH30
G3HMV7	G3HHR3
G3HN14	G3HIM1
G3HN88	G3HK90
G3HNJ3	G3HKG9
G3HNT9	G3HKQ7
G3HNY5	G3HKZ1
G3HQL6	G3HLS2
G3HQM6	G3HLT3
G3HQY2	G3HLV6
G3HR08	G3HM03
G3HRK0	G3HMA1
G3HRK9	G3HMG4
G3HSE4	G3HMQ0
G3HSF3	G3HMV7
G3HSL4	G3HN14
G3HSM3	G3HN88
G3HSX8	G3HNJ3
G3HTE5	G3HNT9
G3HTG9	G3HNY5
G3HU28	G3HQL6
G3HU51	G3HQM6
G3HW06	G3HQP8
G3HWC3	G3HQY2
G3HWE4	G3HR08

G3HWE7	G3HRK0
G3HWJ3	G3HRK9
G3HX39	G3HSF3
G3HXF7	G3HSL4
G3HYB7	G3HSM3
G3HYJ9	G3HSX8
G3HZ42	G3HTE5
G3I015	G3HTG9
G3I1H5	G3HU10
G3I1P5	G3HU28
G3I1V3	G3HV83
G3I1Y9	G3HW06
G3I255	G3HWC3
G3I278	G3HWE4
G3I2H0	G3HWE7
G3I2K6	G3HWJ3
G3I2M1	G3HX39
G3I3G8	G3HXF7
G3I3H2	G3HYB7
G3I3U5	G3HYJ9
G3I3X4	G3HZ42
G3I3Y6	G3I015
G3I4D4	G3I064
G3I4E8	G3I129
G3I4H6	G3I1H5
G3I4W7	G3I1P5
G3I5L3	G3I1V3
G3I5T9	G3I1Y9
G3I5Z5	G3I255
G3I6T1	G3I278
G3I877	G3I2H0
G3I8P7	G3I2K6
G3I8R9	G3I2M1
G3I952	G3I3G8
G3I973	G3I3H2
G3I9F0	G3I3U5
G3I9G7	G3I3X4

G3IAI6	G3I3Y6
G3IAQ0	G3I4D4
G3IAX3	G3I4E8
G3IBH0	G3I4H6
G3IBK2	G3I4W7
G3IDD4	G3I5L3
G3IDL7	G3I5T9
G3IDM2	G3I5Z5
G3IDN7	G3I6T1
G3IDT6	G3I877
G3IEF1	G3I8P7
G3IEU2	G3I8R9
G3IF52	G3I952
G3IFE7	G3I973
G3IG05	G3I9F0
G3IGQ2	G3IAI6
G3IGQ3	G3IAQ0
G3IH63	G3IAX3
G3IHH6	G3IBH0
G3IHY5	G3IBK2
G3IIE7	G3IDD4
G3IIT8	G3IDL7
G3IKC3	G3IDM2
G3IKH9	G3IDN7
G3IKN5	G3IDT6
G3IKQ9	G3IEF1
G3ILF3	G3IEU2
G3ILK7	G3IF52
G3ILN5	G3IF62
G3INC5	G3IF80
G3IPD7	G3IFE7
Q9EPP7	G3IG05
	G3IGQ2
	G3IGQ3
	G3IH63
	G3IHH6
	G3IHY5

G3IIE7
G3IIT8
G3IKC3
G3IKH9
G3IKN5
G3IKQ9
G3ILF3
G3ILK7
G3ILN5
G3INC5
G3IPD7
Q9EPP7
